# MIPP-Seq: ultra-sensitive rapid detection and validation of low-frequency mosaic mutations

**DOI:** 10.1186/s12920-021-00893-3

**Published:** 2021-02-12

**Authors:** Ryan N. Doan, Michael B. Miller, Sonia N. Kim, Rachel E. Rodin, Javier Ganz, Sara Bizzotto, Katherine S. Morillo, August Yue Huang, Reethika Digumarthy, Zachary Zemmel, Christopher A. Walsh

**Affiliations:** 1 Division of Genetics and Genomics, Department of Pediatrics, Boston Children’s Hospital, Center for Life Sciences 15062, 300 Longwood Avenue, BCH3150, Boston, MA 02115 USA; 2 Allen Discovery Center for Human Brain Evolution, Boston Children’s Hospital, Harvard Medical School, Boston, MA USA; 3 Department of Pathology, Brigham and Women’s Hospital, Boston, MA USA; 4 Howard Hughes Medical Institute, Chevy Chase, MD 20815 USA; 5 Departments of Pediatrics and Neurology, Harvard Medical School, Boston, MA USA; 6 Program in Biological and Biomedical Sciences, Harvard University, Boston, MA USA

**Keywords:** Mosaic, Somatic, Validate, Sequencing, Variation

## Abstract

**Background:**

Mosaic mutations contribute to numerous human disorders. As such, the identification and precise quantification of mosaic mutations is essential for a wide range of research applications, clinical diagnoses, and early detection of cancers. Currently, the low-throughput nature of single allele assays (e.g., allele-specific ddPCR) commonly used for genotyping known mutations at very low alternate allelic fractions (AAFs) have limited the integration of low-level mosaic analyses into clinical and research applications. The growing importance of mosaic mutations requires a more rapid, low-cost solution for mutation detection and validation.

**Methods:**

To overcome these limitations, we developed Multiple Independent Primer PCR Sequencing (MIPP-Seq) which combines the power of ultra-deep sequencing and truly independent assays. The accuracy of MIPP-seq to quantifiable detect and measure extremely low allelic fractions was assessed using a combination of SNVs, insertions, and deletions at known allelic fractions in blood and brain derived DNA samples.

**Results:**

The Independent amplicon analyses of MIPP-Seq markedly reduce the impact of allelic dropout, amplification bias, PCR-induced, and sequencing artifacts. Using low DNA inputs of either 25 ng or 50 ng of DNA, MIPP-Seq provides sensitive and quantitative assessments of AAFs as low as 0.025% for SNVs, insertion, and deletions.

**Conclusions:**

MIPP-Seq provides an ultra-sensitive, low-cost approach for detecting and validating known and novel mutations in a highly scalable system with broad utility spanning both research and clinical diagnostic testing applications. The scalability of MIPP-Seq allows for multiplexing mutations and samples, which dramatically reduce costs of variant validation when compared to methods like ddPCR. By leveraging the power of individual analyses of multiple unique and independent reactions, MIPP-Seq can validate and precisely quantitate extremely low AAFs across multiple tissues and mutational categories including both indels and SNVs. Furthermore, using Illumina sequencing technology, MIPP-seq provides a robust method for accurate detection of novel mutations at an extremely low AAF.

## Background

Traditional genetic sequencing methodologies such as whole genome (WGS) and whole exome (WES) sequencing have focused on the important contribution of germline mutations which are present in all cells throughout the human body. However, recent studies have shown numerous examples of mutations occurring after fertilization (i.e.*,* postzygotic mutations), which are only present in a fraction of cells within the body. Postzygotic mutations, or mosaic mutations, have been heavily studied in cancers where clinical diagnostic testing of tumor and blood samples are becoming a standard practice due to improved detection sensitivities [1, 2]. However, the clinical importance of mosaic mutations extends beyond cancer with roles throughout a wide range of neurodevelopmental, overgrowth, and hematological disorders [3–6]. For example, in patients with focal epilepsy, somatic mutations can occur predominately in the brain region where the seizures originate and, thus, are often undetectable using standard germline genomic analyses [3, 4, 7]. As such, improved methods for detecting and validating somatic mutations is essential for clinical testing in these patients.

Furthermore, genetic testing of cell-free DNA (e.g., fetal and tumor) allows for early detection of disease, tracking recurrence in cancers, and even non-invasive prenatal genetic testing where mutations of the fetus are detected in a pregnant mother’s blood [8, 9]. Recent studies have demonstrated that screening for mutations in circulating tumor or cell-free DNA can allow for the early detection of recurring cancers [10–17]. Therefore, rapid and precise assessment of patient or cancer- specific mutational AAFs could provide important clinical benefits for families [10, 11, 13, 17]. Finally, mosaic mutations in healthy individuals are associated with normal development and aging and are, therefore, a powerful tool for understanding how cells divide and form complex organs like the human brain [18, 19].

The rapid advancements in sequencing technologies allow for the detection of genetic mutations present at low alternative allelic fractions (AAF, i.e.*,* ratio of DNA fragments carrying the mutation to those harboring the reference allele) [7, 11, 20–22]. Yet, despite their important role in both clinical and research settings, the analyses of mosaic mutations have yet to be broadly implemented due to significant challenges related to the sensitivity, false positives, accuracy, and the precision of the assessed AAFs [23, 24]. These challenges are often confounded by the inability to directly assess tissues with the highest AAFs, as is the case with neural tissue, or by limited or degraded DNA samples (e.g., cell free DNA) [25–28].

While germline mutations are relatively easy to detect from small amounts of DNA using a range of techniques such as WES, WGS, targeted gene panels, and traditional Sanger sequencing, the AAF of a mosaic mutation will depend on the given tissue, cell type, and the stage in development at which the mutation arose [22, 27]. Traditional WGS and WES in both the research and clinical diagnostic settings are optimized to identify germline events but lack the sequencing depth to robustly detect and quantitate low-AAF variants [23]. However, recent improvements in targeted sequencing allow for the detection of mutations down to 0.1% AAF [6, 29]. While strategies such as molecular barcoding, increased read depth, and reduced use of PCR mitigate sequencing-induced errors [20], the number of false positive low AAF mutations remains higher than germline detection. Therefore, validation of mosaic alleles is often essential, but challenging due to assay costs, throughput, and sensitivity limitations.

The challenge for validating or quantitating low AAFs is multifaceted, spanning sequencing platforms, inherent error rates of polymerases, and locus-specific hurdles. Each of these result in additional errors and skewing of AAFs, which can mask or alter the detected AAF in each assay [30–33]. The utilization of PCR to amplify the genomic loci without inducing additional mutations and maintain the original AAFs has been improved using modified polymerases with proofreading capabilities and, in some cases, unique molecular barcodes for each DNA fragment. Beyond the PCR step, errors can occur during sequencing on both the Illumina and Ion Torrent platforms [20, 31]. For example, in one study, the Ion Torrent had an error rate of ~ 0.05% for SNVs but ~ 1.5% for insertions and deletions (indels), while the Illumina MiSeq had 0.1% errors for SNVs and 0.7% for indels [34]. Beyond technical errors, skewed AAFs, false negatives, and false positives from allelic imbalances due to inherent differences in the genome content around a mutation must all be considered when interpreting AAFs. Even more, additional mutations, repeat content, DNA methylation, and copy number changes can have dramatic impacts on AAFs, resulting in the commonly recognized issue of allelic dropout [33]. While primers are commonly designed to avoid areas with known genetic polymorphisms, the assays remain susceptible to allelic skewing from ultra-rare or private alleles and other loci specific causes of allelic imbalance.

In recent years several approaches have been utilized for validating and quantifying mosaic alleles including pyrosequencing [2, 35, 36] and bacterial cloning followed by Sanger sequencing of hundreds or thousands of individual bacterial colonies to measure a single mutation [28, 37, 38]. These methods, while accurate and robust, were often cost-prohibitive, less scalable to large numbers of mutations, and less sensitive for mutations below 5% AAF. Allele-specific digital droplet PCR (ddPCR) assays improved sensitivity to measure AAFs through counting mutation positive and negative DNA fragments in thousands of droplets using a single amplicon [21, 39] and is routinely considered a gold standard in both research and clinical settings. While the ddPCR assay accurately detects AAFs below 0.5%, it requires the development of a custom assay, validation, and optimization to assess large numbers of droplets in each reaction [39]. Recently, blocker displacement amplification (BDA) [40] was shown to robustly detect low AAF variants down to 0.1%. This technology allows for multiplexing using different florescent color probes, differing amplicon band size by gel electrophoresis, or DNA sequencing. The authors of BDA note that such a strategy substantially improves on the costs and complexity of developing assays for detecting low AAF alleles [40]. However, despite their success, ddPCR and BDA remain limited by scalability, availability of unique fluorescent color channels, allelic dropout, and the ability to design allele-specific primers or blockers, which is more challenging in repetitive regions and for small indels.

The growing consensus that mosaic mutations underlie a wide range of clinical phenotypes spanning from cancer risk to severe neurodevelopmental and overgrowth conditions suggests that a robust method for detection, quantification, and validation of variant alleles is essential. Multiple Independent Primer PCR Sequencing (MIPP-Seq) aims to mitigate the previously stated limitations for assessing mosaic mutations. Our strategy relies on the power of analyzing multiple independent, nonoverlapping amplicons over a targeted locus. Independent amplicon analyses markedly reduce the impact of allelic dropout, amplification bias, PCR-induced, and sequencing artifacts, while achieving the highest sensitivity to accurately detect ultra-low allelic fractions down to at least 0.05% AAF. As described below, our method allows for additional improvements to further improve accuracy using molecular barcoding and improved purification processes for both the detection and validation of novel and known alleles.

## Methods

### Primer design

For complete protocol, see Additional file 1: Methods. At least three unique sets of primers were designed for each mutation using BedTools [41] getfasta with the reference genome (hg19) to extract the flanking sequence around each mutation so that the mutation is located at different positions within each of the three sequences. Next, common alleles are masked, along with the targeted mutation and flanking 5bps on each site using the bedtools maskfasta tool. The masked multi-fasta file containing all sequences for targeted alleles are input into BatchPrimer [42] webtool to design primers for each sequence. Primers are designed to an average TM of 60C, with a minimum of 59 and maximum of 62C. The amplicon length is dependent on the specific mutation and DNA sources, for example difficult to map region may have longer products while degraded DNA samples may require shorter amplicons. In general, to ensure that all primers are likely unique and of similar amplicon length, amplicons have a target length of 225–300 bp in length. The primer sequences are checked by BLAT and in-silico PCR to ensure both their unique amplificon in the genome and that the primer binding sites do not overlap between any set of primers. The final set of primers are then uniquely barcoded using 10nt barcodes and if desired, an additional 10nt UMI is added. Finally, Ion Torrent or Illumina specific adapter sequences are appended to the forward and reverse primers.

### Library preparation

Previously isolated DNA, extracted from whole blood or postmortem human brain specimens [43], from deidentified samples were utilized for all analyses. The brain tissues were obtained from Lieber Institute for Brain Development, the NIH NeuroBioBank, and the Autism BrainNet. All specimens were deidentified and all research was approved by the institutional review board of Boston Children’s Hospital.

For the standard, single step PCR method of MIPP-Seq, PCR was performed using 20 cycles on a 25ul reaction mix containing either 25 or 50 ng of input DNA sample, Phusion Hot-Start polymerase, dNTPs, HC-Buffer, and the primers. For initial testing, 30 cycles of enrichment were used to ensure only a single amplicon is produced. The high-sensitivity method modifies this process by reduction of the PCR cycling to 5 and the incorporation of 0.1 uL of 0.4 mM biotin-14-dCTP (Thermofisher) into the reaction mix. Biotinylated PCR amplicons are captured by adding 5ul of washed Streptavidin MyOne beads resuspended in 25 ul of 2X binding and washing buffer. The mixture is incubated at room temperature with gentle mixing for 30 min and placed on a 96-well magnetic plate. The liquid was removed, and the beads were washed one time with 1X binding and washing buffer. Then beads are then resuspended in 25 ul PCR reaction mixture containing custom primers which preserve the original UMI sequences, Phusion Hot-Start polymerase, dNTPs, and HC-Buffer. The biotin labeled product was amplified with an additional 20 cycles of enrichment before the beads were removed. Enriched products were further purified using 0.7X AMPure XP magnetic beads (Beckman Coulter).

### QC and Variant calling

Purified library pools are analyzed for enrichment efficiency and the complete removal of primers through by either the Agilent Bioanalyzer Hi-sensitivity chip or the Agilent D1000 ScreenTape System. The concentration was determined using the Quant-iT dsDNA high sensitivity assay kit (Thermofisher). Pools were diluted to a final concentration of 100 pM prior to sequencing on 430 chips for the Ion Torrent S5.

Raw unmapped bam files were obtained for each run and were processed using our custom analyses pipeline. First, all BAMs were converted to fastq using bedtool’s bamtofastq tool [41]. Next, the samples were demultiplexed using the unique 15nt barcodes (5nt of the primer and 10nt index) using FASTX toolkit’s fastx_barcode_splitter (-bol -mismatches 3) resulting in fastq files for each primer set. If the allele being tested in an SNV, indel correction was performed using Pollux [44] (-n false -d false -h true -s false -f false). Then, barcode and quality trimming were performed using the cutadapt [45] tool (-u 10 -q 10). Finally, all samples are aligned to the reference genome using default settings in BWA-mem with local indel realignment being performed with GATK 3.7 IndelRealigner [46] (-greedy 1200 -maxReads 2,000,000 -maxInMemory 1,500,000) with indels present in gnomAD being used as a reference. Finally, primer binding sites were removed using the bamclipper tool [47] with default settings.

All BAMs were for the sensitivity analyses were randomly downsampled using Samtools [48] and were indexed for variant calling. Variants were called across the length of each amplicon using Samtools mPileup with the settings: q = 20, Q = 20. The resulting VCFs were parsed into files containing the flanking 50nt positions on each side of the variant and a separate file for the allele of interest. Allelic positions within these flanking regions with additional known germline mutations were excluded to avoid artificially inflating the error rates.

### Assessment of AAF

The measured AAF of mutations were calculated using the following steps (Additional file 2: Figure S1). The AAF at the variant position was extracted from the VCF for each of the amplicons, for example, 3 unique primers resulted in 3 unique measurements of the AAF. The average and 95% confidence intervals were calculated to determine the precision of the variant calls. The significance of measured AAFs were determined using the primer-specific error rates. These background error rates and standard deviations of mutations, representing the chances of generating a mutational artifact, were calculated using the average allele frequencies across the 100 bases flanking the assessed mutations in each of the amplicons. Finally, the significance of assessed AAFs against the background error rates were assessed using both the 95% confidence intervals and a t-test. As a comparison, above steps are also performed on the raw data which was not error-corrected using Pollux.

### Modification for Illumina platform

The PRNP gene was tiled with PCR primers so that all coding regions were covered by at least three unique primer sets each having unique primer binding sites. All primers were designed so that the maximum amplicon length was less than 285 bp, including the primers. Standard Illumina adapter sequences and 5 nucleotide UMIs were added to the forward and reverse primers. All primers were ordered in individual tubes to avoid the risk of cross contamination during the printing process.

## Results

**Fig. 1:**
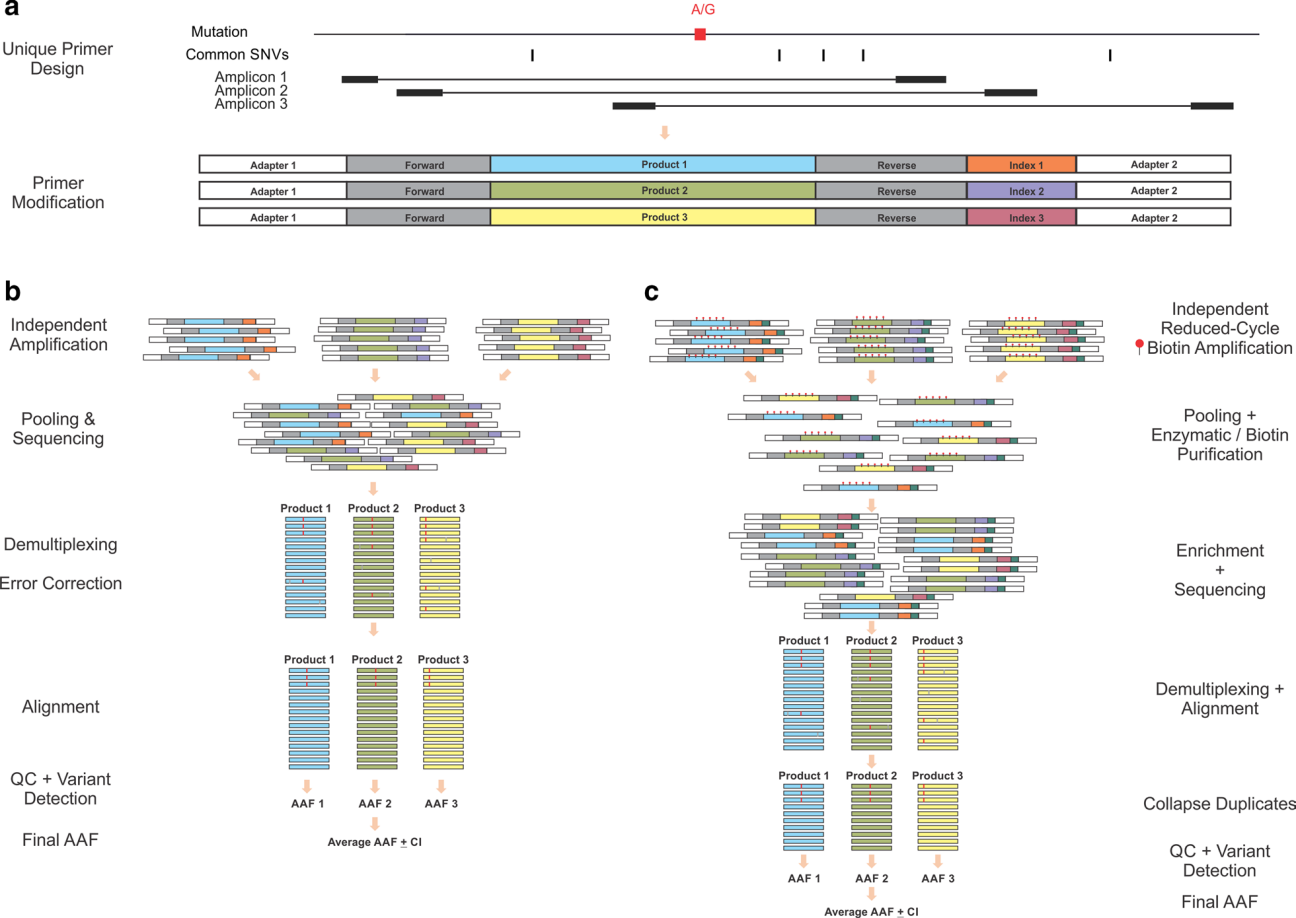
Overview of MIPP-Seq method. **a** Design and modification of multiple unique primers to generate amplicons spanning targeted mutation of interest using either **b** the standard single-step or C) 2-step UMI-containing MIPP-Seq workflows for detection and validation of mutations

Here we describe Multiple Independent Primer PCR Sequencing (MIPP-Seq) which substantially increases the throughput and sensitivity for the detection and validation of mosaic mutations (Fig. 1). Our method utilizes multiple sets of primers designed to avoid overlapping primer binding sites and common causes of allelic dropout such as additional genetic variants. MIPP-Seq offers a flexible and robust solution for both the identification of novel mutations and assessments of AAFs of known mutations in one or more samples. Unlike existing methods such as ddPCR, MIPP-Seq often requires little to no optimization after primer design and has broad sensitivity regardless of DNA source (e.g., blood and brain derived), concentration, and nucleotide context. Here we demonstrate the robust sensitivity of MIPP-Seq to detect and validate mosaic mutations using the Ion Torrent S5 platform and a modified version for the detection of novel alleles using Illumina sequencing.

### Sensitivity and detection limits of MIPP-Seq

**Fig. 2:**
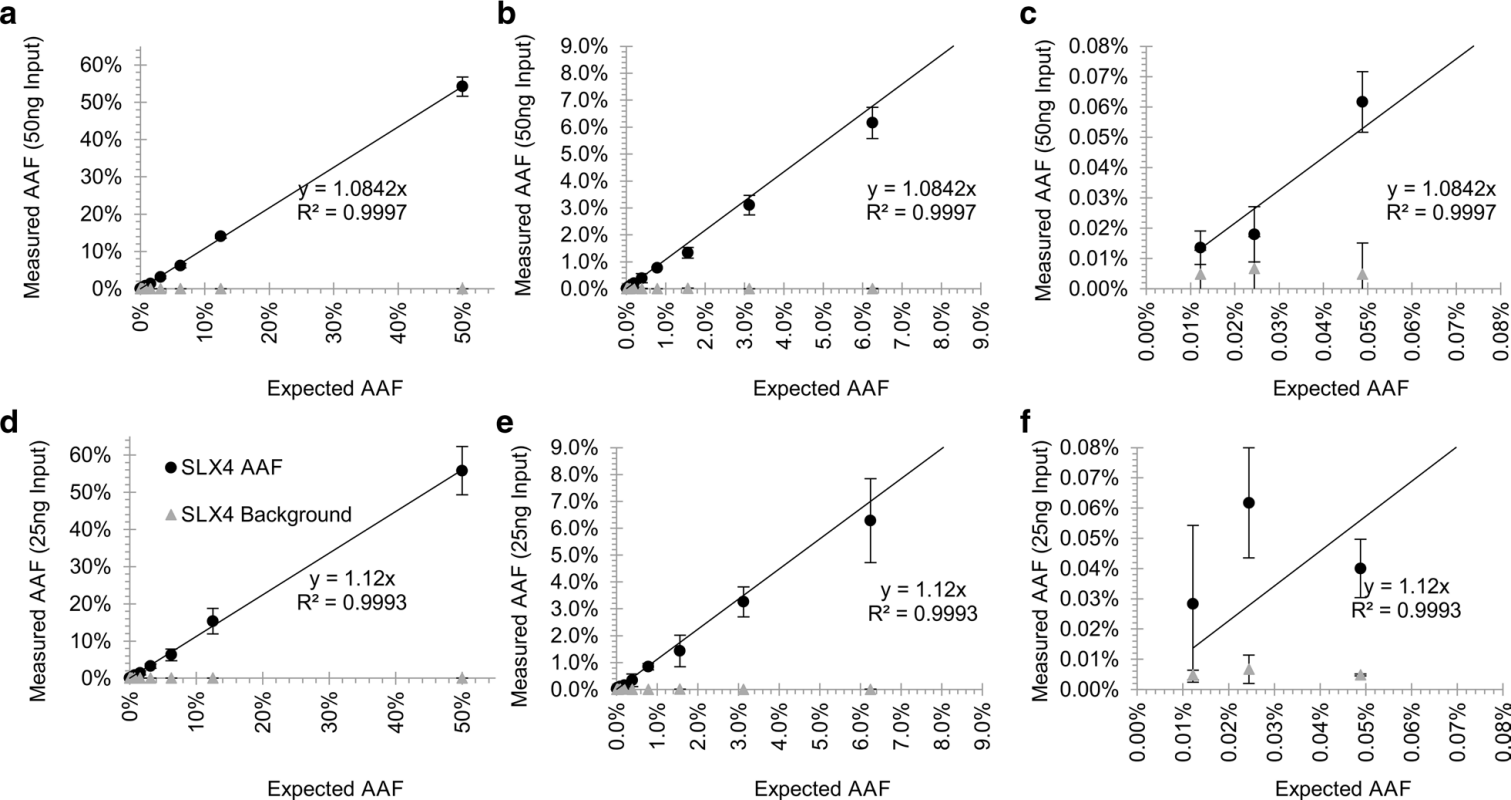
Minimal impact on sensitivity for reduced PCR DNA input for Mutation 1. Sensitivity to measure the AAF and background error through a dilution curve of a polymorphism (Mutation 1) using **a** 50 ng 0.01% to 50% AAF and data subsets with AAFs **b** less than 9% and **c** less than 0.08%. Reduction of DNA input to 25 ng with **d** 0.01% to 50% AAF and data subsets with AAFs **e** less than 9% and **f** less than 0.08%

MIPP-Seq’s sensitivity limits were assessed through analyses of serial dilutions of genomic samples with three known germline mutations using three unique amplicons per allele (Additional file 1: Table S1). The dilutions generated known AAFs ranging from 50% down to 0.01%. Furthermore, MIPP-Seq was assessed on germline heterozygous mutations, yielding expected measurements of 50% AAF with great precision (Additional file 3: Figure S2). The measured AAFs were linearly correlated with the expected AAFs down to 0.01% (R^2^ > 0.99), though as expected, individual AAFs do vary amongst individual primers (R^2^ > 0.98). Even more, MIPP-Seq accurately detects AAFs as low as 0.01% with all three assessed mutational dilution curves when using 50 ng of genomic DNA, although for significant detection above the amplicon-specific error rates, AAFs were typically required to be at least 0.025% (Fig. 2a–c, Additional file 4: Fig. S3, Additional file 5: Fig. S4). Surprisingly, MIPP-Seq achieved a 100% sensitivity for detection of alleles down to 0.01% AAF with all alleles being detected by at least 1 of the amplicons (Fig. 2c, Additional file 4: Fig. S3, Additional file 5: Fig. S4). The measured AAF of the 2048-fold dilution was ascertained to be 0.0136% ± 0.006% while the background error rate remained substantially lower at 0.007% ± 0.004%. As DNA quantity is often limited in clinical settings, we compared the impact on sensitivity of reduced DNA input from 50 to 25 ng [~ 3800 cells [49]]. Surprisingly, AAFs down to 0.025% remained detectable with 25 ng DNA (Fig. 2d–f, Additional file 4: Fig. S3, Additional file 5: Fig. S4), though with less precision (0.028% ± 0.0025% AAF), suggesting that increased DNA input is important to maintain the quantitative assessment of alleles below 0.1% AAF.

Furthermore, another key factor of a quantitative measurement is its precision, which is also partially built into MIPP-seq through assessment of the confidence intervals across the multiple primer sets for a given mutation. In most instances, primers for a given mutation yield extremely similar AAFs, resulting in very small standard deviations compared to the measure AAFs (Fig. 2a–c, Additional file 4: Fig. S3, Additional file 5: Fig. S4). A large standard deviation can occur due to allelic dropout in one of the three primers in a set but can often be identified by the presence of an additional nearby genetic variant.

**Fig. 3:**
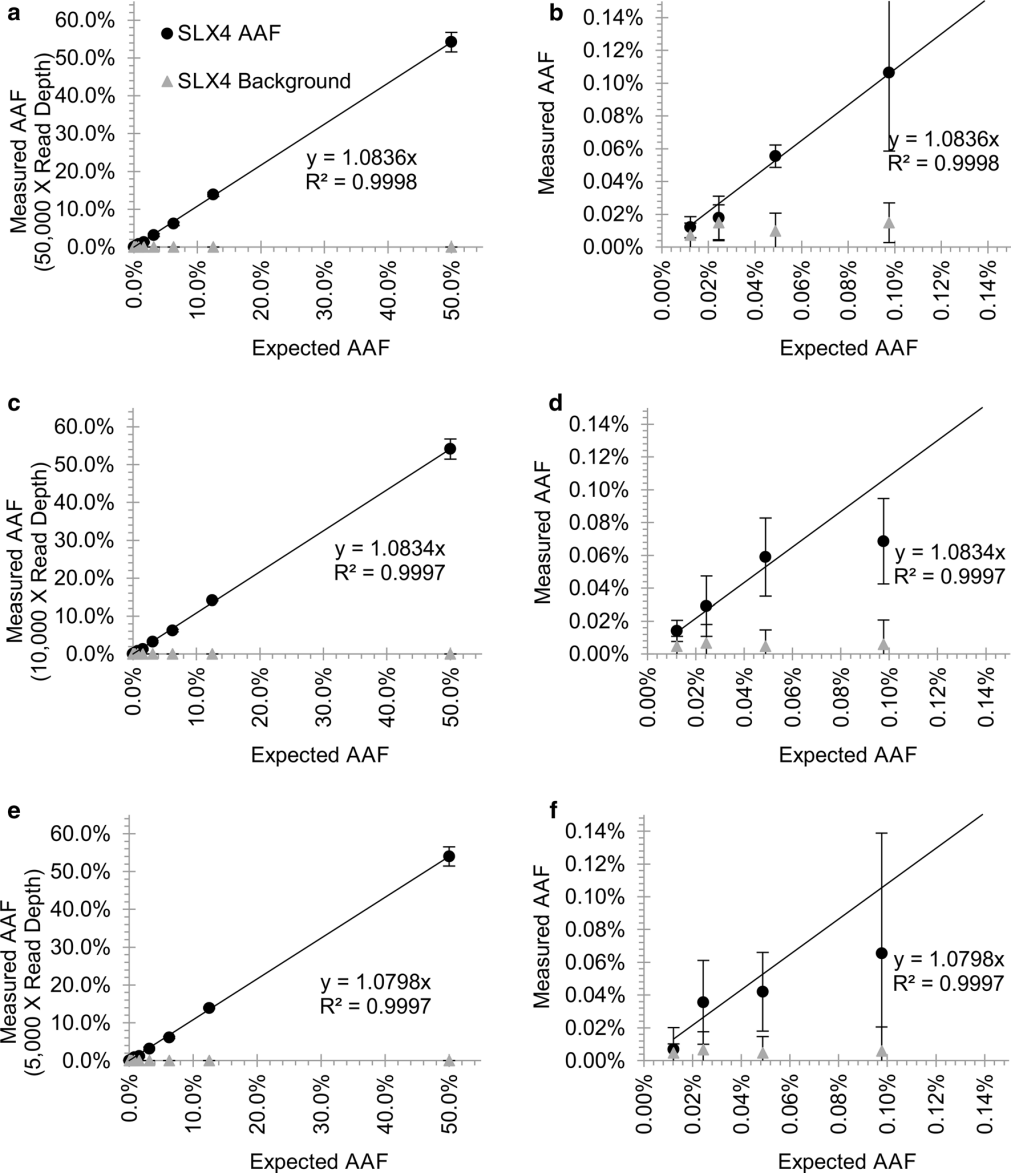
Impact of read depth on sensitivity of AAF assessments for Mutation 1. Reduction of initial maximum read depth from 50,000X for detection of alleles from **a** 50% to **b** 0.025% to **c**, **d** 10,000X and **e**, **f** 5000X

Read depth directly impacted the precision of the AAF measurements. Mapped BAM files for each amplicon were randomly sampled to generate datasets containing read depths from 5,000 to 150,000X coverage (Fig. 3, Additional file 6: Fig. S5, Additional file 7: Fig. S6). While increased depths had little impact on amplicon error rates, depths of at least 10,000X were able to accurately measure AAFs down to 0.1%, while deeper coverage beyond that gave only minimal further accuracy. However, accurate measurement of AAFs below 0.1% were improved with depths of 50,000X to distinguish real alleles from background errors. Overall, we find a strong correlation of AAFs measured across a wide range of read depths, suggesting that the largest factor in assessing AAFs below 0.1% was providing sufficient input DNA and achieving enough sequencing depth to distinguish artifacts from true calls.

We further extended our assessment of error rates and the potential for false positive allele calls by performing similar sequencing on DNA samples lacking mutations. As expected, none of the variant alleles were detectable, with only the typical background error rate being detected, which is often not the same allele as the mutation, supporting the specificity of this method.

### Low nucleotide error rates

As the utility of MIPP-Seq relies on overcoming the previously described sources of quantification error, we evaluated error rates across the assessed mutations. Our reduced PCR cycling conditions with a high-fidelity polymerase (Phusion HS, ThermoFisher) are estimated to result in an error rate of 8.8 × 10^–6^ at any given nucleotide position (ThermoFisher PCR Fidelity Calculator). Indel-associated errors were reduced using Pollux [44], a recent error modeling algorithm that screens for and corrects many indel-associated errors. Pollux reduced the already low nucleotide error frequency (0.01% AAF ± 0.0012%) by nearly 30% (0.007% ± 0.0012%, Additional file 8: Fig. S7), allowing for mutations at extremely low AAFs to be distinguished from background sequencing and PCR-induced artifacts (Figs. 2, 3, Additional file 4: Fig. S3, Additional file 5: Fig. S4, Additional file 6: Fig. S5, Additional file 7: Fig. S6). While Pollux reduced the error rates, raw and final AAFs of targeted mutations remained highly correlated (R^2^ = 1, Additional file 9: Fig. S8).

### Precise assessment of AAFs in tissue-derived DNA

**Fig. 4:**
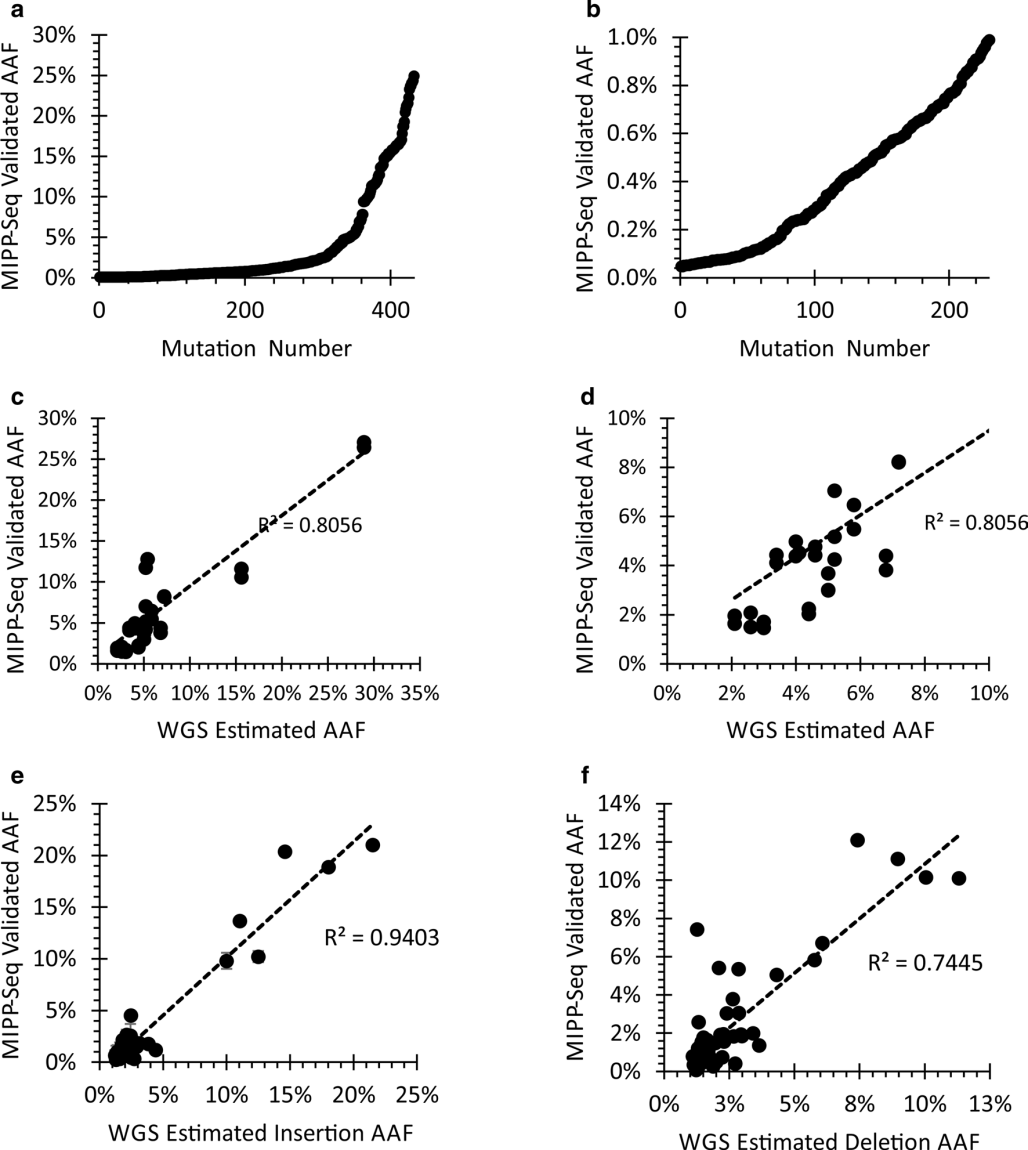
Validation of mosaic alleles detected from neural tissues. MIPP-seq validated AAFs from **a** 432 alleles up to 30%AAF with a subset of alleles **b** with AAFs below 1%. Correlation of AAFs previously detected by 200X WGS sequencing in brain tissue and MIPP-seq for AAFs less than **c** 30% and **d** 10%. Strong correlation of AAFs of WGS and MIPP-seq for both **e** insertions and **f** deletions detected and validated in brain derived DNA samples

We further validated the ability of MIPP-Seq to assess alleles in other tissues using 482 previously identified somatic SNVs from brain-derived DNA in healthy individuals (432 SNVs, Fig. 4a, b, Kim et. al. and Ganz et. al. unpublished data) and those with Autism Spectrum Disorder (50 SNVs, ASD, Fig. 4c, d) [25, 50]. As expected, somatic mutations were readily detectable in brain-derived samples with AAFs down to 0.05%. Even more, mosaic mutations can be properly phased with nearby germline polymorphisms (Additional file 10: Fig. S9). While most AAFs were similar to the originally detected rates, the dissimilar AAFs were typically associated with low coverage in the original sequencing platform or a single outlier amplicon with allelic dropout caused by a germline polymorphism (Additional file 11: Fig. S10). The occurrence of allelic dropout highlights the importance of using multiple primers when studying mosaic and germline alleles.

### Robust validation for low AAF insertions/deletions

The elevated sequencing-induced errors around homopolymers in Ion Torrent sequencing data combined with limited PCR duplicate information may reduce the sensitivity to precisely quantitate some ultra-low AAF indels (< 0.05% AAF) [34, 44]. Even more, the Pollux software is known to overcorrect for indels [44, 51] and has difficulty distinguishing rare indels from artifacts. Despite these limitations, we assessed MIPP-Seq performance on indels occurring at a wide range of AAFs from 1 to 30% and 1 to 21 base pairs in length, including 40 insertions and 60 deletions previously identified using 200X whole genome sequencing [43]. Even more importantly, we do not identify these mutations in control DNA (Additional file 12: Fig. S11), where at these sites we find very low error rates for indels (0.010% ± 0.05%) supporting that even the single base indels are not being introduced by PCR or the Ion Torrent platform. These data suggest a sensitivity to accurately quantitate AAFs of indels down to 0.05%. Despite being detected using only a few reads in the WGS data, we find a strong correlation between the predicted AAFs in the WGS and the measured values by MIPP-Seq (Fig. 4e, f; R^2^ = 0.75 deletions and R^2^ = 0.94 for insertions).

To further improve our sensitivity for low AAFs, we developed a modified protocol (Fig. 1b) with an initial low-cycle PCR containing biotinylated dCTP (~ 25% of a cytosines), or biotinylated primers, with unique molecular indexes (UMIs), to uniquely tag all PCR products in the first 10 cycles. After purification using either streptavidin capture or enzymatic digestion (see methods), all reactions are further amplified by a common primer that maintains the UMI signature, effectively tagging all PCR duplicates from the 2^nd^ round of PCR. The incorporation of biotin into the PCR product did not impact the overall measured AAFs, but slightly reduced the error rate (0.0023% ± 0.0011% AAF), possibly due to the ability to perform better purification and the use of a common primer for the majority of the amplifications. These suggest that a 2-step UMI approach for MIPP-Seq might be valuable in situations requiring reduced error rates for ultra-low AAFs, removal of PCR duplicates, or consensus-based allele calling.

### MIPP-Seq accurately detects mutations when modified for Illumina-based sequencing platforms

**Fig. 5:**
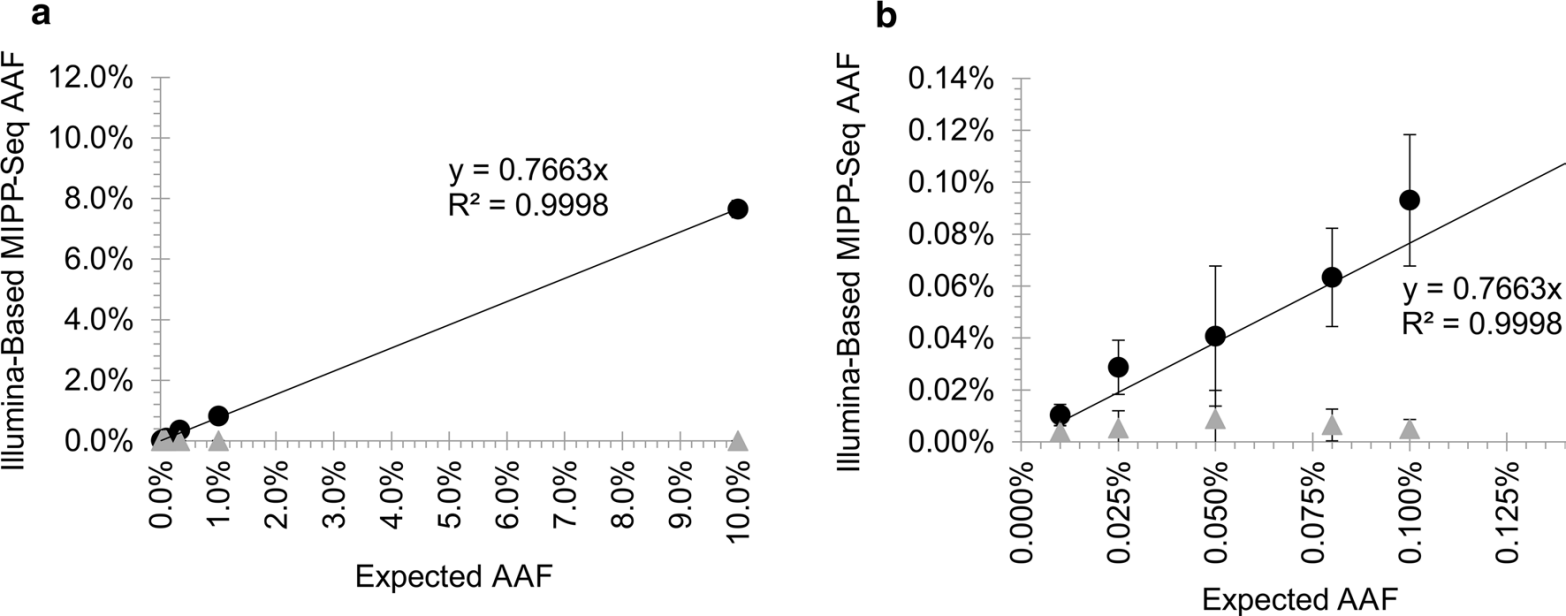
Validation of Illumina modified MIPP-seq to allow for sensitive detection of mosaic alleles. Sensitivity curve for detection of serially diluted mutation (black filled circles) versus low error rate (grey triangles) for AAFs **a** up to 10% and **b** below 0.125%

The increased sensitivity of the MIPP-Seq approach can be further applied for the detection of novel ultra-low AAFs variants with Illumina-based sequencing. In order to determine the sensitivity of an Illumina-compatible MIPP-Seq approach to quantify and detect new alleles, we developed a 2-step PCR approach where overlapping unique primer were designed to target each locus. All targeted bases were covered by four independent amplicons, each containing Illumina sequencing adapters and UMIs. Using a 2-step PCR approach, we prepared sequencing libraries for a dilution series with a known mutation at eight AAFs from 0.01 to 10% AAF. Despite performing 2 sequential rounds of PCR amplification, we accurately quantified the AAFs of targeted mutation down to at least 0.025% with an average read depth of just 13,766X, with background error rates comparable to those of our Ion Torrent based approach (Fig. 5a, b). Even more, we find that while sequencing artifacts may occur in each amplicon due to polymerase errors, sequencing platforms, etc.; the errors detected were random and unlikely to occur across all primers targeting the loci. Therefore, by requiring that a novel mutation be detectable above background in most amplicons (3 of 4 amplicons), potential false positive mutations at very low AAFs can be substantially reduced. In the targeted loci here, we observed no false positive calls across the regions targeted by the set of 4 amplicons. These data suggest that Illumina-modified MIPP-Seq can accurately detect mutations down to at least 0.025% AAF, suggesting a possible option for improved accurate measurement of AAFs of novel alleles in targeted sequencing platforms.

## Discussion

Mosaic mutations contribute to a wide range of genetic disorders beyond cancers including those impacting hematological [52], muscular [53], cardiovascular [54, 55], and neurological [4, 25, 26, 50, 56, 57] systems, but their identification and validation often remain challenging. Here we describe MIPP-Seq as a comprehensive method for the detection, quantification, and validation of known and novel genetic mutations across a wide range of AAFs and tissue types. MIPP-Seq markedly reduces the impact of allelic dropout, amplification bias, and induced artifacts (e.g., PCR and sequencing induced), while achieving a high sensitivity to accurately detect ultra-low allelic fractions below 0.05% regardless of tissue origin. Furthermore, MIPP-Seq allows for additional improvements to further improve accuracy through incorporations of molecular barcoding, improved purification processes, and compatibility for additional sequencing platforms.

Prior studies have demonstrated the validation of low AAF alleles using ultra-deep amplicon sequencing using single sets of PCR primers [7, 25, 50, 57]. However, allelic dropout and artifacts (e.g., PCR- and sequencing platform-induced) can reduce the sensitivity of single amplicon strategies, detected AAFs and possibly result in both false negative calls as well as skewed AAFs. MIPP-Seq overcomes the limitations of powerful assays such as ddPCR and BDA [40], which often utilize a single set of primers and probes, by using multiple unique barcoded primers for independent assessments of AAF, amplicon-specific error rates, and allelic imbalances. Furthermore, the costs associated with the highly scalable MIPP-Seq approach can be tenfold lower than ddPCR due to the combination of minimal optimization, ability to assess hundreds of mutations per sequencing run, use of standard primer synthesis, and a streamlined analytical pipeline. Thus, MIPP-Seq provides a scalable and rapid strategy for consistently precise estimation of AAFs which is broadly applicable to clinical and research studies of mosaic and germline mutations in human disease [4, 26, 29, 50, 53, 54, 56, 57] and normal development [18, 20, 25, 37]. In particular, the ability to utilize MIPP-Seq on multiple sequencing platforms and to simultaneously assess hundreds of variants with little optimization allows for a substantial reduction in the cost to validate any given allele. Therefore, MIPP-Seq provides an ideal solution that will enable clinical diagnostics to expand the breadth of available mosaic testing more broadly in families.

Another challenge of genomic studies involves testing of low quality or degraded DNA samples such as those from cell-free [8, 58, 59] and circulating tumor [1, 10, 15, 17, 30, 58] specimens. We demonstrate the feasibility of utilizing MIPP-Seq in different DNA sources with little to no additional modifications beyond adjusting the amplicon size. The flexibility of MIPP-Seq to utilize a wide range of amplicon sizes and multiplexing reactions enables both personalized and disease specific screening of cell-free and/or circulating tumor specimens from patients to monitor the improvements gained due to therapies or to detect the early recurrence of cancers [10–14, 58, 60]. Such multiplex batches would also enable rapid and highly sensitive validation of variants from deep sequencing gene panels and WGS [18, 25, 61]. Furthermore, a similar approach could be applied to prenatal testing for both the detection of known mutations and for screening of novel mutations [8, 9, 59].

Even more, as MIPP-Seq relies on PCR, it can be feasibly utilized to fill other needs in the research and clinical communities where quantitative measurements are essential for extremely low AAFs. For example, recent studies have highlighted the importance of understanding bacterial and viral loads in the microbiome [62, 63] and wastewater [64–68]. However, the low bacterial or viral DNA content and the presence of large amount of external DNA contamination (i.e.*,* human, animal, insect DNA) complicate such analyses [69]. MIPP-seq allows individual viral or bacterial genotypes to be quantified without the need to sequence DNA from other contaminants. Even more, MIPP-seq could allow for the detection of mutational profiles within essential viral domains which are targeted by vaccines, thereby allowing for earlier detection of new viral mutations. Finally, it is feasible to apply MIPP-Seq to sample types such as RNA and cDNA, including viral, with minimal modifications.

Finally, the application of MIPP-Seq for novel mutation detection could provide a much higher resolution and quantitative strategy to detect novel mosaic alleles across entire genes or regions such as the mitochondrial genome, which accounts for numerous severe disorders [70–72]. Genetic diagnoses of disorders of disorders involving the mitochondrial genome are particularly challenging due to heteroplasmy [73, 74], which results in variable allelic fractions across tissues. To overcome this challenge, numerous sequencing methodologies have been developed with detection limitations ranging from 0.1 to 10% AAF [73, 74]. However, due to elevated false positive and negative rates for AAFs < 1.5% of many of these sequencing approaches require AAFs to be above 3% for accurate detection [73, 74]. The highest sensitivity approach, ddPCR, allows for precise assessment of a single known mutation, but lacks the ability to screen the entire mitochondrial genome for novel mutations. The application of MIP-Seq toward mitochondrial genetic testing could future improve upon these approaches, and potentially provide additional genetic diagnosis.

## Conclusions

The importance of mosaic mutations in both genetic research and clinical diagnostic testing are reliant on high quality detection and validation of alleles. However, to date, the costs and complexity of such validation have limited the expansion of clinical diagnostic testing and large validation of research studies. Here we describe Multiple Independent PCR Sequencing (MIPP-Seq) as a flexible method for both low and high throughput detection and validation of mosaic mutations. This scalable platform can be applied to both small and large projects at a fraction of the cost and time as leading methods like ddPCR. MIPP-Seq leverages the power of individual analyses of multiple unique PCR amplicons from independent reactions to identify novel mutations or quantification of AAFs of known mutations. We demonstrate that the highly sensitive MIPP-Seq can validate and precisely quantitate extremely low AAFs across a wide range of tissues and mutational categories including both indels and SNVs. Together, this approach can be applied to a wide range of processes including research and clinical allele validation, cell-free DNA, and clinical testing and screening in oncology patients.

## Supplementary Information

**Additional file 1.** Methods.

**Additional file 2: Fig S1.** Variant allelic fraction assessment across multiple primers. A) The AAF of the targeted mutation is compared to the background error rate of 50nts flanking each side of the mutation and B) the assessed rates are averaged across all unique primers for the mutation.

**Additional file 3: Fig S2.** Example of validated heterozygous germline mutation. The targeted SNV was identified in A) sequencing reads for all 3 unique primers, allowing for B) the measured AAF of 50%.

**Additional file 4: Fig S3.** Impact on sensitivity for reduced PCR DNA input for Mutation 2. Sensitivity to measure the AAF and background error through a dilution curve of a polymorphism (Mutation 2) using A) 50ng 0.01% to 50% AAF and data subsets with AAFs B) less than 9% and C) less than 0.08%. Reduction of DNA input to 25ng with D) 0.01% to 50% AAF and data subsets with AAFs E) less than 9% and F) less than 0.08%.

**Additional file 5: Fig S4.** Impact on sensitivity for reduced PCR DNA input for Mutation 3. Sensitivity to measure the AAF and background error through a dilution curve of a polymorphism (Mutation 3) using A) 50ng 0.01% to 50% AAF and data subsets with AAFs B) less than 9% and C) less than 0.08%. Reduction of DNA input to 25ng with D) 0.01% to 50% AAF and data subsets with AAFs E) less than 9% and F) less than 0.08%.

**Additional file 6: Fig S5.** Impact of read depth on sensitivity of AAF assessments for Mutation 2. Reduction of initial maximum read depth from 50,000X for detection of alleles from A) 50% to B) 0.025% to C) & D) 10,000X and E) & F) 5,000X.

**Additional file 7: Fig S6.** Impact of read depth on sensitivity of AAF assessments for Mutation 3. Reduction of initial maximum read depth from 50,000X for detection of alleles from A) 50% to B) 0.025% to C) & D) 10,000X and E) & F) 5,000X.

**Additional file 8: Fig S7.** Reduction of background average per base error rate through error correction.

**Additional file 9: Fig S8.** Strong correlation of AAFs before and after error correction by Pollux algorithm. A) Correlation of raw AAFs, those detected from data prior to error correction, with the final AAFs (i.e., post-error correction), with B) a subset of data below 7% AAF also showing a strong correlation.

**Additional file 10: Fig S9.** Mosaic mutation in cis with germline polymorphism. A) Analysis of a mosaic point mutation revealed a germline polymorphism in 2 of the 3 amplicons. Amplicons 1 and 2 (B &C) covered both the germline and mosaic event and confirmed the cis arrangement, while the 3rd amplicon D) did not include the germline event.

**Additional file 11: Fig S10.** Detection of allele dropout masking germline event. A germline mutation was targeted by A) 3 unique sets of primers. Mapped sequencing data for B) amplicon 1 and C) amplicon 2 yielded the expected 50% AAF mutation and identified a common polymorphism nearby and located in the binding site of the D) third primer, interfering with binding and resulting in a dramatically skewed AAF.

**Additional file 12: Fig S11.** Comparison of AAFs detected for indels in cases vs controls. Indels were validated using MIPP-seq on the case DNA sample contained a suspected indel and a different control DNA sample lacking the indel. All indels validated by MIPP-seq exhibited a significantly higher AAF in the case (black filled circles) vs control DNA (grey triangles) A) and B).

## Data Availability

The datasets used and/or analyzed during the current study are available from the corresponding author on request. Data generated in this study can be downloaded from GEO database by using access number: GSE165780. The hg19 reference genome used in this study is available to download from http://hgdownload.soe.ucsc.edu/goldenPath/hg19/bigZips/hg19.fa.gz.

## Abbreviations

AAF: Alternate allelic fraction
BDA: Blocker displacement amplification
CI: Confidence interval
ddPCR: Digital droplet PCR
Indel: Insertion or deletion
MIPP-Seq: Multiple Independent Primer PCR Sequencing
PCR: Polymerase chain reaction
SNV: Single nucleotide variant
TM: Melting temperature
UMI: Unique Molecular Index
WES: Whole exome sequencing
WGS: Whole Genome sequencing
