# Supplementary material for: MIPP-Seq: ultra-sensitive rapid detection and validation of low-frequency mosaic mutations
Source: BMC Med Genomics. 2021 Feb 12;14:47. doi: 10.1186/s12920-021-00893-3 (PMC7881461; doi:10.1186/s12920-021-00893-3)
Supplement: Supplementary file 1 — Additional file 1. Methods. [file 12920_2021_893_MOESM1_ESM.docx]

**Supplementary Information**

**Table of Contents**

- 1. **Supplementary Methods – Pages 2-5**
  2. **Supplementary Table 1 – Pages 6-7**

**Supplementary** **Methods**

**Primer Design:**

Unique primers are designed to prevent sharing binding sites and avoiding known mutations in the individual and general population but are within 250 nucleotides of the target mutation. Once designed, unique 10nt barcodes are appended to the reverse primer, along with the standard Ion Torrent platform specific adapters. Optionally, Illumina adapters and/or 10nt molecular barcodes can be appended to the primers to improved sensitivity or usage on the Illumina platform.

**Mosaic Alleles: 3 Unique Primers**

1. Generation of Fasta files for regions of interest
   1. Create bed file of all mutations in excel
   2. 3 sets of regions for each mutation
      1. +/- 150bp
      2. - 175bp + 50bp
      3. -50bp + 175bp
      4. Save as bed file (example.bed)
   3. Create new masked genome in which all variants of interest are masked, including 5bp up and downstream
      1. Create bed file of all variants with 5bp up/downstream
         1. If variant = chr1:23456778
            1. Bed file : chr1 23456773 23456783
            2. Upload to orchestra
         2. Mask genome using this bed file: on orchestra:
            1. module load seq/BEDtools

*bedtools maskfasta -fi ucsc.hg19.fa -bed variants_to_mask.bed -fo ucsc.hg19.variantmasked.fa*

- 1. Use bedtools to get fasta sequence from masked reference genome (masked with validation variations and common variants)

*bedtools getfasta -fi ucsc.hg19.variantmasked.fa -bed example.bed > example.fa*

- 1. Designing primers - BatchPrimer
     1. Min TM = 59
     2. Max TM = 62
     3. Min amplicon length = 225
     4. Max amplicon length = 300
  2. Once complete, open link from email to view primers
     1. Download CSV or TAB file with selected primers
     2. Any regions missing 3 primers will need additional designing (either changing settings in the program of manually in Primer3Plus)
     3. Check all primers by BLAT to ensure they are unique and primer binding sites miss known mutations

**For Germline mutations or screening larger # of mutations:**

1. Follow above steps to design primers, but only select 1 primer per mutation by using +/- 150bp region

**Finalizing primers for ordering:**

1. Open Ion_Torrent_Design_Template.xlsx file
2. Paste all primers into the sheet as indicated by example in file
   1. Select if UMIs needed
   2. This will add a 10nt barcode on the reverse primer, a 10nt molecular barcode, along with P and A adapters for the Ion Torrent. (if primers are unique, it will use the primer as the barcode to allow for shorter primers)
3. Order primers from using standard desalting

**Library Preparation Process:**

The primers designed above amplify targets using reduced cycling and minimal amounts of DNA and are sequenced on either the Ion Torrent S5 or Illumina MiSeq platform for ultra-deep coverage. This optimized process allows for independent analyses of each primer pair, determination of error rates bases on amplicon-specific error rates (*i.e*., level of PCR and sequencing induced artifacts at across the amplicon), identification of allelic imbalances from additional mutations affecting primer binding or chromatin structure, and the assessment of the variation in AAF among primers. Increasing input from 25ng genomic DNA to 50ng increases sensitivity for mutations at or below 0.1% AAF.

**Version 1: for Alleles with AAFs above 0.1%**

**PCR Reaction Conditions: (creates sequencing libraries in single PCR step)**

1. PCR Mix (25ulrxn)
   1. 5X HF Buffer 5ul
   2. 10mM dNTPs 0.5ul
   3. 10uM F/R primer mix 2.5
   4. DNA (25-50ng) 2ul
   5. Phusion Pol HF 0.25ul
   6. H2O 14.75

98C 30”

98C 10” \\\\\\

62C 20” 20 X

72C 30” //////

72C 10’

4C hold

**Purification of Above Reactions:**

1. Purification
   1. Pool 5ul from each rxn (they are all barcoded at this point)
   2. Use ThermoFisher MagJet purification kit or any other kit that removes products <100bp in length
      1. Resuspend in **20uL** of water, pipette to mix (less is better is using few # of samples)
      2. Incubate 2 minutes
      3. Place on magnet 2minutes
      4. Remove all eluted DNA into new plate
      5. Run 1ul product on Tapestation or Bioanalyzer to confirm quality
   3. Determine concentration by picoGreen measurement
2. Dilute to 100pM concentration
3. Send for 400bp sequencing on Ion Torrent S5

**Version 2: for Alleles with AAFs above 0.025%**

**PCR Reaction # 1 Conditions: (adds biotin tag and UMIs to all products)**

1. PCR Mix (25ulrxn) with **Biotin**
   1. 5X HF Buffer 5ul
   2. 10mM dNTPs 0.5ul
   3. 10uM F/R primer mix 2.5
   4. DNA (50ng) 2ul
   5. Phusion Pol HF 0.25ul
   6. 0.4mM Biotin-14-dCTP 0.1ul
   7. H2O 14.65

98C 30”

98C 10” \\\\\\

62C 20” 8X

72C 30” //////

72C 10’

4C hold

**Purification of Above Reactions:**

**SELECT 1 OF 2 OPTIONS FOR STEP 2: Biotin Capture or ExoSap-IT Treatment**

**Option 1: Biotin-Capture:**

1. Biotin Binding per rxn:
   1. Wash 4ul of streptavidin beads 2 times with 1X Binding-Washing buffer (B&W)
   2. Resuspend in 25ul of 2X B&W buffer
   3. Transfer 25ul beads to 25ul PCR mixture
      1. Incubate at RT 20’ with mixing
      2. Bind to magnet and remove liquid
   4. Add 100ul 1X B&W buffer
      1. Mix & place on magnet and remove liquid
      2. Repeat wash 1x
   5. Wash 1X with 200ul Water
   6. Resuspend beads in 28ul water

**Option 2: ExoSap-IT:**

1. ExoSap-It treatment to remove primers
   1. Add 8ul of exo-sap-it to 20ul of PCR product
   2. Place in thermocycler
      1. 15min at 37C
      2. 15min at 80C
      3. 4C hold

**PCR Reaction # 2: either #1 or #2 depending on if using biotin or exosapit: (enriches libraries while maintaining UMIs)**

1. Enrichment PCR - 50ul rxn
   1. Streptavidin-bound DNA 28ul
   2. 5X HF Buffer 10ul
   3. 10mM dNTPs 1ul
   4. 10uM custom primer mix 2.5
   5. Phusion Pol HF 0.5ul
   6. H2O 8
2. Enrichment PCR if using exoSapIt - 50ul rxn
   1. Exosap-it treated rxn 28ul
   2. 5X HF Buffer 6ul
   3. 10mM dNTPs 1ul
   4. 10uM custom primer mix 2.5
   5. Phusion Pol HF 0.5ul
   6. H2O 12

98C 30”

98C 10” \\\\\\

62C 20” 25X

72C 30” //////

72C 10’

4C hold

**Purification of Above Reactions:**

1. Purification
   1. Pool each rxn (they are all barcoded at this point)
   2. Use ThermoFisher MagJet purification kit or any other kit that removes products <100bp in length
      1. Resuspend in **20uL** of water, pipette to mix (less is better is using few # of samples)
      2. Incubate 2 minutes
      3. Place on magnet 2minutes
      4. Remove all eluted DNA into new plate
      5. Run 1ul product on Tapestation or Bioanalyzer to confirm quality
   3. Determine concentration by picoGreen measurement
2. Dilute to 100pM concentration
3. Send for 400bp sequencing on Ion Torrent S5

**Supplementary Table 1. Mutations and primers used for sensitivity analyses.**

| **Chr** | **Allele** | **Forward** | **Reverse** |
| --- | --- | --- | --- |
| X | 153579431 | CCTCTCTATGGGCAGTCGGTGATCAGGGCCTCACCTTGGTC | CCATCTCATCCCTGCGTGTCTCCGACTCAGttaacggacgCGCCAGATGGGTAAGTGC |
| X | 153579431 | CCTCTCTATGGGCAGTCGGTGATCTGTGACATAGCACTCCTCCAG | CCATCTCATCCCTGCGTGTCTCCGACTCAGtccggcttacTGCAAATCAGTGGCTCTCC |
| X | 153579431 | CCTCTCTATGGGCAGTCGGTGATAGGCTGGCTGGTTGACCT | CCATCTCATCCCTGCGTGTCTCCGACTCAGtctcattcagCTCCCTTCCTGCCACCTG |
| 12 | 46321441 | CCTCTCTATGGGCAGTCGGTGATAATCACACTCCATAGGTATCATTTCA | CCATCTCATCCCTGCGTGTCTCCGACTCAGgcggtcatacACATGTGATACTTTTGGGAATGAAG |
| 12 | 46321441 | CCTCTCTATGGGCAGTCGGTGATTTCATTCATTTGTTTAAGATCAGCA | CCATCTCATCCCTGCGTGTCTCCGACTCAGtaggacgttcCTTCTGAACACCAAATTGGAAA |
| 12 | 46321441 | CCTCTCTATGGGCAGTCGGTGATTCAATGTGTGTTTTAGGCAACTC | CCATCTCATCCCTGCGTGTCTCCGACTCAGacgacgcaacTGTTAAGAGCCCAGAGGTTCA |
| X | 153594210 | CCTCTCTATGGGCAGTCGGTGATAGGGGGACATGCAAGACA | CCATCTCATCCCTGCGTGTCTCCGACTCAGcttctcggacGGGGCCCCTACTCTTTGA |
| X | 153594210 | CCTCTCTATGGGCAGTCGGTGATGCGAGCTCTTCCGAAGGT | CCATCTCATCCCTGCGTGTCTCCGACTCAGcattgccgttCTCGCAGCCCCTACACTG |
| X | 153594210 | CCTCTCTATGGGCAGTCGGTGATGTTGACCCTGTGGGCAGA | CCATCTCATCCCTGCGTGTCTCCGACTCAGcgagccagaaTGACTGCCCTCTGCTGTG |
| 16 | 3639306 | CCTCTCTATGGGCAGTCGGTGATTCCTCTGGGTAGTGCAGCTT | CCATCTCATCCCTGCGTGTCTCCGACTCAGtgaggacggcAGTGACGATGAGCAGGAGGT |
| 16 | 3639306 | CCTCTCTATGGGCAGTCGGTGATCAGAGCCGAATTCAGAAAGC | CCATCTCATCCCTGCGTGTCTCCGACTCAGgcctgcgcagGCCAATTCCCATTGACCA |
| 16 | 3639306 | CCTCTCTATGGGCAGTCGGTGATGGGGTGGTGTCCAGGAGT | CCATCTCATCCCTGCGTGTCTCCGACTCAGgttgacgtctCCAAGCTTCCTGAACCAGAC |
| X | 153599770 | CCTCTCTATGGGCAGTCGGTGATCATTTTGAGGCGCGAGAA | CCATCTCATCCCTGCGTGTCTCCGACTCAGgagatcgattCTAGTGGGGGCATTCCAA |
| X | 153599770 | CCTCTCTATGGGCAGTCGGTGATGAGGCAGGGAGCAGAGGT | CCATCTCATCCCTGCGTGTCTCCGACTCAGagttcgagccCTCTAGGGCGCGTTTCCT |
| X | 153599770 | CCTCTCTATGGGCAGTCGGTGATCCTTTAAATGCGGGAGGAG | CCATCTCATCCCTGCGTGTCTCCGACTCAGctcaggctcaTCAGCCTTTCCTCGCTCTA |
| 18 | 21453038 | CCTCTCTATGGGCAGTCGGTGATGAGCAGGAAGGGCAGGTATAA | CCATCTCATCCCTGCGTGTCTCCGACTCAGggcaatataaTCCACATAACTCGCTTGCAG |
| 18 | 21453038 | CCTCTCTATGGGCAGTCGGTGATCTGGCACAGGCTGACTCAT | CCATCTCATCCCTGCGTGTCTCCGACTCAGggtactcatgGAACTGTAGCCCAGACACTGC |
| 18 | 21453038 | CCTCTCTATGGGCAGTCGGTGATGGATGCCTCCAGCAGTGA | CCATCTCATCCCTGCGTGTCTCCGACTCAGtctggttcaaACAAAGCTGGAAACTCTTCCCTA |
| X | 153587777 | CCTCTCTATGGGCAGTCGGTGATAGCCTCATAAGGGATGTACTCG | CCATCTCATCCCTGCGTGTCTCCGACTCAGgtcctataagCCAACAAGCCCAACAAGTTC |
| X | 153587777 | CCTCTCTATGGGCAGTCGGTGATCCTTGAAAGGACTGCCTGAG | CCATCTCATCCCTGCGTGTCTCCGACTCAGgtcagcctccGAATGACCGGCTGTCTGTTT |
| X | 153587777 | CCTCTCTATGGGCAGTCGGTGATCTCCTCACCTGGCACTTGAT | CCATCTCATCCCTGCGTGTCTCCGACTCAGttcaagctcgAAAGTGGCACCACCAACAA |
| 19 | 44571260 | CCTCTCTATGGGCAGTCGGTGATAGAGCCCACACAGGAGAGAG | CCATCTCATCCCTGCGTGTCTCCGACTCAGgtaccagcgcCTTGTAGCGCTTCCCACAGT |
| 19 | 44571260 | CCTCTCTATGGGCAGTCGGTGATATCAGCGAGTCCACACTGG | CCATCTCATCCCTGCGTGTCTCCGACTCAGtcctattcggAGCTTCTTTCCACAATCCTCA |
| 19 | 44571260 | CCTCTCTATGGGCAGTCGGTGATTTGAATCATAAGAGACTCCATTGC | CCATCTCATCCCTGCGTGTCTCCGACTCAGgccagcgattCTGTACCCCATAAATATGTACAACACT |
| X | 153579431 | CCTCTCTATGGGCAGTCGGTGATCAGGGCCTCACCTTGGTC | CCATCTCATCCCTGCGTGTCTCCGACTCAGacctagactgCGCCAGATGGGTAAGTGC |
| X | 153579431 | CCTCTCTATGGGCAGTCGGTGATCTGTGACATAGCACTCCTCCAG | CCATCTCATCCCTGCGTGTCTCCGACTCAGactggttcgcTGCAAATCAGTGGCTCTCC |
| X | 153579431 | CCTCTCTATGGGCAGTCGGTGATAGGCTGGCTGGTTGACCT | CCATCTCATCCCTGCGTGTCTCCGACTCAGccatattaggCTCCCTTCCTGCCACCTG |
| 12 | 46321441 | CCTCTCTATGGGCAGTCGGTGATAATCACACTCCATAGGTATCATTTCA | CCATCTCATCCCTGCGTGTCTCCGACTCAGgctcgtcagcACATGTGATACTTTTGGGAATGAAG |
| 12 | 46321441 | CCTCTCTATGGGCAGTCGGTGATTTCATTCATTTGTTTAAGATCAGCA | CCATCTCATCCCTGCGTGTCTCCGACTCAGcgtaatgacgCTTCTGAACACCAAATTGGAAA |
| 12 | 46321441 | CCTCTCTATGGGCAGTCGGTGATTCAATGTGTGTTTTAGGCAACTC | CCATCTCATCCCTGCGTGTCTCCGACTCAGccggcgctgaTGTTAAGAGCCCAGAGGTTCA |
| X | 153594210 | CCTCTCTATGGGCAGTCGGTGATAGGGGGACATGCAAGACA | CCATCTCATCCCTGCGTGTCTCCGACTCAGcgcgaagataGGGGCCCCTACTCTTTGA |
| X | 153594210 | CCTCTCTATGGGCAGTCGGTGATGCGAGCTCTTCCGAAGGT | CCATCTCATCCCTGCGTGTCTCCGACTCAGgaaccgcagaCTCGCAGCCCCTACACTG |
| X | 153594210 | CCTCTCTATGGGCAGTCGGTGATGTTGACCCTGTGGGCAGA | CCATCTCATCCCTGCGTGTCTCCGACTCAGttggcagagaTGACTGCCCTCTGCTGTG |
| 16 | 3639306 | CCTCTCTATGGGCAGTCGGTGATTCCTCTGGGTAGTGCAGCTT | CCATCTCATCCCTGCGTGTCTCCGACTCAGgcatctctgcAGTGACGATGAGCAGGAGGT |
| 16 | 3639306 | CCTCTCTATGGGCAGTCGGTGATCAGAGCCGAATTCAGAAAGC | CCATCTCATCCCTGCGTGTCTCCGACTCAGttggaccgcaGCCAATTCCCATTGACCA |
| 16 | 3639306 | CCTCTCTATGGGCAGTCGGTGATGGGGTGGTGTCCAGGAGT | CCATCTCATCCCTGCGTGTCTCCGACTCAGgcagaacgtcCCAAGCTTCCTGAACCAGAC |
| X | 153599770 | CCTCTCTATGGGCAGTCGGTGATCATTTTGAGGCGCGAGAA | CCATCTCATCCCTGCGTGTCTCCGACTCAGaacttcgagcCTAGTGGGGGCATTCCAA |
| X | 153599770 | CCTCTCTATGGGCAGTCGGTGATGAGGCAGGGAGCAGAGGT | CCATCTCATCCCTGCGTGTCTCCGACTCAGgctcctagagCTCTAGGGCGCGTTTCCT |
| X | 153599770 | CCTCTCTATGGGCAGTCGGTGATCCTTTAAATGCGGGAGGAG | CCATCTCATCCCTGCGTGTCTCCGACTCAGtatctagcttTCAGCCTTTCCTCGCTCTA |
| 18 | 21453038 | CCTCTCTATGGGCAGTCGGTGATGAGCAGGAAGGGCAGGTATAA | CCATCTCATCCCTGCGTGTCTCCGACTCAGgagtattggcTCCACATAACTCGCTTGCAG |
| 18 | 21453038 | CCTCTCTATGGGCAGTCGGTGATCTGGCACAGGCTGACTCAT | CCATCTCATCCCTGCGTGTCTCCGACTCAGcctgagctcaGAACTGTAGCCCAGACACTGC |
| 18 | 21453038 | CCTCTCTATGGGCAGTCGGTGATGGATGCCTCCAGCAGTGA | CCATCTCATCCCTGCGTGTCTCCGACTCAGcaggcgagtaACAAAGCTGGAAACTCTTCCCTA |
| X | 153587777 | CCTCTCTATGGGCAGTCGGTGATAGCCTCATAAGGGATGTACTCG | CCATCTCATCCCTGCGTGTCTCCGACTCAGgcaggcagagCCAACAAGCCCAACAAGTTC |
| X | 153587777 | CCTCTCTATGGGCAGTCGGTGATCCTTGAAAGGACTGCCTGAG | CCATCTCATCCCTGCGTGTCTCCGACTCAGgcgtcgatacGAATGACCGGCTGTCTGTTT |
| X | 153587777 | CCTCTCTATGGGCAGTCGGTGATCTCCTCACCTGGCACTTGAT | CCATCTCATCCCTGCGTGTCTCCGACTCAGcgatgattatAAAGTGGCACCACCAACAA |
| 19 | 44571260 | CCTCTCTATGGGCAGTCGGTGATAGAGCCCACACAGGAGAGAG | CCATCTCATCCCTGCGTGTCTCCGACTCAGgacggctggcCTTGTAGCGCTTCCCACAGT |
| 19 | 44571260 | CCTCTCTATGGGCAGTCGGTGATATCAGCGAGTCCACACTGG | CCATCTCATCCCTGCGTGTCTCCGACTCAGggagcctgagAGCTTCTTTCCACAATCCTCA |
| 19 | 44571260 | CCTCTCTATGGGCAGTCGGTGATTTGAATCATAAGAGACTCCATTGC | CCATCTCATCCCTGCGTGTCTCCGACTCAGcctgactgctCTGTACCCCATAAATATGTACAACACT |
| X | 153579431 | CCTCTCTATGGGCAGTCGGTGATCAGGGCCTCACCTTGGTC | CCATCTCATCCCTGCGTGTCTCCGACTCAGacggctgacgCGCCAGATGGGTAAGTGC |
| X | 153579431 | CCTCTCTATGGGCAGTCGGTGATCTGTGACATAGCACTCCTCCAG | CCATCTCATCCCTGCGTGTCTCCGACTCAGtaaccatagcTGCAAATCAGTGGCTCTCC |
| X | 153579431 | CCTCTCTATGGGCAGTCGGTGATAGGCTGGCTGGTTGACCT | CCATCTCATCCCTGCGTGTCTCCGACTCAGtcttgccttcCTCCCTTCCTGCCACCTG |
| 12 | 46321441 | CCTCTCTATGGGCAGTCGGTGATAATCACACTCCATAGGTATCATTTCA | CCATCTCATCCCTGCGTGTCTCCGACTCAGttcttagattACATGTGATACTTTTGGGAATGAAG |
| 12 | 46321441 | CCTCTCTATGGGCAGTCGGTGATTTCATTCATTTGTTTAAGATCAGCA | CCATCTCATCCCTGCGTGTCTCCGACTCAGtcatctcattCTTCTGAACACCAAATTGGAAA |
| 12 | 46321441 | CCTCTCTATGGGCAGTCGGTGATTCAATGTGTGTTTTAGGCAACTC | CCATCTCATCCCTGCGTGTCTCCGACTCAGtctccgctcgTGTTAAGAGCCCAGAGGTTCA |
| X | 153594210 | CCTCTCTATGGGCAGTCGGTGATAGGGGGACATGCAAGACA | CCATCTCATCCCTGCGTGTCTCCGACTCAGtgccatatgcGGGGCCCCTACTCTTTGA |
| X | 153594210 | CCTCTCTATGGGCAGTCGGTGATGCGAGCTCTTCCGAAGGT | CCATCTCATCCCTGCGTGTCTCCGACTCAGtaaggcctctCTCGCAGCCCCTACACTG |
| X | 153594210 | CCTCTCTATGGGCAGTCGGTGATGTTGACCCTGTGGGCAGA | CCATCTCATCCCTGCGTGTCTCCGACTCAGgagtaggccgTGACTGCCCTCTGCTGTG |
| 16 | 3639306 | CCTCTCTATGGGCAGTCGGTGATTCCTCTGGGTAGTGCAGCTT | CCATCTCATCCCTGCGTGTCTCCGACTCAGgcaataagctAGTGACGATGAGCAGGAGGT |
| 16 | 3639306 | CCTCTCTATGGGCAGTCGGTGATCAGAGCCGAATTCAGAAAGC | CCATCTCATCCCTGCGTGTCTCCGACTCAGggcgttgcaaGCCAATTCCCATTGACCA |
| 16 | 3639306 | CCTCTCTATGGGCAGTCGGTGATGGGGTGGTGTCCAGGAGT | CCATCTCATCCCTGCGTGTCTCCGACTCAGccaagaagcgCCAAGCTTCCTGAACCAGAC |
| X | 153599770 | CCTCTCTATGGGCAGTCGGTGATCATTTTGAGGCGCGAGAA | CCATCTCATCCCTGCGTGTCTCCGACTCAGggttacctcgCTAGTGGGGGCATTCCAA |
| X | 153599770 | CCTCTCTATGGGCAGTCGGTGATGAGGCAGGGAGCAGAGGT | CCATCTCATCCCTGCGTGTCTCCGACTCAGctccgccttaCTCTAGGGCGCGTTTCCT |
| X | 153599770 | CCTCTCTATGGGCAGTCGGTGATCCTTTAAATGCGGGAGGAG | CCATCTCATCCCTGCGTGTCTCCGACTCAGctccagagatTCAGCCTTTCCTCGCTCTA |
| 18 | 21453038 | CCTCTCTATGGGCAGTCGGTGATGAGCAGGAAGGGCAGGTATAA | CCATCTCATCCCTGCGTGTCTCCGACTCAGgtcgaggtagTCCACATAACTCGCTTGCAG |
| 18 | 21453038 | CCTCTCTATGGGCAGTCGGTGATCTGGCACAGGCTGACTCAT | CCATCTCATCCCTGCGTGTCTCCGACTCAGtatggacctgGAACTGTAGCCCAGACACTGC |
| 18 | 21453038 | CCTCTCTATGGGCAGTCGGTGATGGATGCCTCCAGCAGTGA | CCATCTCATCCCTGCGTGTCTCCGACTCAGtacctgctagACAAAGCTGGAAACTCTTCCCTA |
| X | 153587777 | CCTCTCTATGGGCAGTCGGTGATAGCCTCATAAGGGATGTACTCG | CCATCTCATCCCTGCGTGTCTCCGACTCAGccgcgaccgaCCAACAAGCCCAACAAGTTC |
| X | 153587777 | CCTCTCTATGGGCAGTCGGTGATCCTTGAAAGGACTGCCTGAG | CCATCTCATCCCTGCGTGTCTCCGACTCAGgttgaacgttGAATGACCGGCTGTCTGTTT |
| X | 153587777 | CCTCTCTATGGGCAGTCGGTGATCTCCTCACCTGGCACTTGAT | CCATCTCATCCCTGCGTGTCTCCGACTCAGtgccaacgcaAAAGTGGCACCACCAACAA |
| 19 | 44571260 | CCTCTCTATGGGCAGTCGGTGATAGAGCCCACACAGGAGAGAG | CCATCTCATCCCTGCGTGTCTCCGACTCAGggattgacctCTTGTAGCGCTTCCCACAGT |
| 19 | 44571260 | CCTCTCTATGGGCAGTCGGTGATATCAGCGAGTCCACACTGG | CCATCTCATCCCTGCGTGTCTCCGACTCAGggacggattcAGCTTCTTTCCACAATCCTCA |
| 19 | 44571260 | CCTCTCTATGGGCAGTCGGTGATTTGAATCATAAGAGACTCCATTGC | CCATCTCATCCCTGCGTGTCTCCGACTCAGtcctccgtcgCTGTACCCCATAAATATGTACAACACT |
| X | 153579431 | CCTCTCTATGGGCAGTCGGTGATCAGGGCCTCACCTTGGTC | CCATCTCATCCCTGCGTGTCTCCGACTCAGagttcatggtCGCCAGATGGGTAAGTGC |
| X | 153579431 | CCTCTCTATGGGCAGTCGGTGATCTGTGACATAGCACTCCTCCAG | CCATCTCATCCCTGCGTGTCTCCGACTCAGtatccattccTGCAAATCAGTGGCTCTCC |
| X | 153579431 | CCTCTCTATGGGCAGTCGGTGATAGGCTGGCTGGTTGACCT | CCATCTCATCCCTGCGTGTCTCCGACTCAGggagagcgcgCTCCCTTCCTGCCACCTG |
| 12 | 46321441 | CCTCTCTATGGGCAGTCGGTGATAATCACACTCCATAGGTATCATTTCA | CCATCTCATCCCTGCGTGTCTCCGACTCAGcggaccttggACATGTGATACTTTTGGGAATGAAG |
| 12 | 46321441 | CCTCTCTATGGGCAGTCGGTGATTTCATTCATTTGTTTAAGATCAGCA | CCATCTCATCCCTGCGTGTCTCCGACTCAGggcaatctccCTTCTGAACACCAAATTGGAAA |
| 12 | 46321441 | CCTCTCTATGGGCAGTCGGTGATTCAATGTGTGTTTTAGGCAACTC | CCATCTCATCCCTGCGTGTCTCCGACTCAGaggattgattTGTTAAGAGCCCAGAGGTTCA |
| X | 153594210 | CCTCTCTATGGGCAGTCGGTGATAGGGGGACATGCAAGACA | CCATCTCATCCCTGCGTGTCTCCGACTCAGgccgttgcctGGGGCCCCTACTCTTTGA |
| X | 153594210 | CCTCTCTATGGGCAGTCGGTGATGCGAGCTCTTCCGAAGGT | CCATCTCATCCCTGCGTGTCTCCGACTCAGaagtacgtcgCTCGCAGCCCCTACACTG |
| X | 153594210 | CCTCTCTATGGGCAGTCGGTGATGTTGACCCTGTGGGCAGA | CCATCTCATCCCTGCGTGTCTCCGACTCAGtggcttaaggTGACTGCCCTCTGCTGTG |
| 16 | 3639306 | CCTCTCTATGGGCAGTCGGTGATTCCTCTGGGTAGTGCAGCTT | CCATCTCATCCCTGCGTGTCTCCGACTCAGctcttccagaAGTGACGATGAGCAGGAGGT |
| 16 | 3639306 | CCTCTCTATGGGCAGTCGGTGATCAGAGCCGAATTCAGAAAGC | CCATCTCATCCCTGCGTGTCTCCGACTCAGcgttcttcaaGCCAATTCCCATTGACCA |
| 16 | 3639306 | CCTCTCTATGGGCAGTCGGTGATGGGGTGGTGTCCAGGAGT | CCATCTCATCCCTGCGTGTCTCCGACTCAGcaacggctgcCCAAGCTTCCTGAACCAGAC |
| X | 153599770 | CCTCTCTATGGGCAGTCGGTGATCATTTTGAGGCGCGAGAA | CCATCTCATCCCTGCGTGTCTCCGACTCAGgcaagtaaccCTAGTGGGGGCATTCCAA |
| X | 153599770 | CCTCTCTATGGGCAGTCGGTGATGAGGCAGGGAGCAGAGGT | CCATCTCATCCCTGCGTGTCTCCGACTCAGgttcatagtcCTCTAGGGCGCGTTTCCT |
| X | 153599770 | CCTCTCTATGGGCAGTCGGTGATCCTTTAAATGCGGGAGGAG | CCATCTCATCCCTGCGTGTCTCCGACTCAGacggcgagccTCAGCCTTTCCTCGCTCTA |
| 18 | 21453038 | CCTCTCTATGGGCAGTCGGTGATGAGCAGGAAGGGCAGGTATAA | CCATCTCATCCCTGCGTGTCTCCGACTCAGgtatggtcggTCCACATAACTCGCTTGCAG |
| 18 | 21453038 | CCTCTCTATGGGCAGTCGGTGATCTGGCACAGGCTGACTCAT | CCATCTCATCCCTGCGTGTCTCCGACTCAGtcggttatccGAACTGTAGCCCAGACACTGC |
| 18 | 21453038 | CCTCTCTATGGGCAGTCGGTGATGGATGCCTCCAGCAGTGA | CCATCTCATCCCTGCGTGTCTCCGACTCAGgcggtcgataACAAAGCTGGAAACTCTTCCCTA |
| X | 153587777 | CCTCTCTATGGGCAGTCGGTGATAGCCTCATAAGGGATGTACTCG | CCATCTCATCCCTGCGTGTCTCCGACTCAGtcctcagtatCCAACAAGCCCAACAAGTTC |
| X | 153587777 | CCTCTCTATGGGCAGTCGGTGATCCTTGAAAGGACTGCCTGAG | CCATCTCATCCCTGCGTGTCTCCGACTCAGaccgttcctgGAATGACCGGCTGTCTGTTT |
| X | 153587777 | CCTCTCTATGGGCAGTCGGTGATCTCCTCACCTGGCACTTGAT | CCATCTCATCCCTGCGTGTCTCCGACTCAGgcctgctcttAAAGTGGCACCACCAACAA |
| 19 | 44571260 | CCTCTCTATGGGCAGTCGGTGATAGAGCCCACACAGGAGAGAG | CCATCTCATCCCTGCGTGTCTCCGACTCAGagcgtaaccaCTTGTAGCGCTTCCCACAGT |
| 19 | 44571260 | CCTCTCTATGGGCAGTCGGTGATATCAGCGAGTCCACACTGG | CCATCTCATCCCTGCGTGTCTCCGACTCAGttgcctgatgAGCTTCTTTCCACAATCCTCA |
| 19 | 44571260 | CCTCTCTATGGGCAGTCGGTGATTTGAATCATAAGAGACTCCATTGC | CCATCTCATCCCTGCGTGTCTCCGACTCAGttattgatctCTGTACCCCATAAATATGTACAACACT |
| X | 153579431 | CCTCTCTATGGGCAGTCGGTGATCAGGGCCTCACCTTGGTC | CCATCTCATCCCTGCGTGTCTCCGACTCAGtacgctcggaCGCCAGATGGGTAAGTGC |
| X | 153579431 | CCTCTCTATGGGCAGTCGGTGATCTGTGACATAGCACTCCTCCAG | CCATCTCATCCCTGCGTGTCTCCGACTCAGcaatccaaggTGCAAATCAGTGGCTCTCC |
| X | 153579431 | CCTCTCTATGGGCAGTCGGTGATAGGCTGGCTGGTTGACCT | CCATCTCATCCCTGCGTGTCTCCGACTCAGtcgtagctatCTCCCTTCCTGCCACCTG |
| 12 | 46321441 | CCTCTCTATGGGCAGTCGGTGATAATCACACTCCATAGGTATCATTTCA | CCATCTCATCCCTGCGTGTCTCCGACTCAGcgctcatcgcACATGTGATACTTTTGGGAATGAAG |
| 12 | 46321441 | CCTCTCTATGGGCAGTCGGTGATTTCATTCATTTGTTTAAGATCAGCA | CCATCTCATCCCTGCGTGTCTCCGACTCAGtccgttcattCTTCTGAACACCAAATTGGAAA |
| 12 | 46321441 | CCTCTCTATGGGCAGTCGGTGATTCAATGTGTGTTTTAGGCAACTC | CCATCTCATCCCTGCGTGTCTCCGACTCAGcggccaggctTGTTAAGAGCCCAGAGGTTCA |
| X | 153594210 | CCTCTCTATGGGCAGTCGGTGATAGGGGGACATGCAAGACA | CCATCTCATCCCTGCGTGTCTCCGACTCAGcaacctatctGGGGCCCCTACTCTTTGA |
| X | 153594210 | CCTCTCTATGGGCAGTCGGTGATGCGAGCTCTTCCGAAGGT | CCATCTCATCCCTGCGTGTCTCCGACTCAGcgtaatctcaCTCGCAGCCCCTACACTG |
| X | 153594210 | CCTCTCTATGGGCAGTCGGTGATGTTGACCCTGTGGGCAGA | CCATCTCATCCCTGCGTGTCTCCGACTCAGatatcgcgacTGACTGCCCTCTGCTGTG |
| 16 | 3639306 | CCTCTCTATGGGCAGTCGGTGATTCCTCTGGGTAGTGCAGCTT | CCATCTCATCCCTGCGTGTCTCCGACTCAGtcaatatctgAGTGACGATGAGCAGGAGGT |
| 16 | 3639306 | CCTCTCTATGGGCAGTCGGTGATCAGAGCCGAATTCAGAAAGC | CCATCTCATCCCTGCGTGTCTCCGACTCAGatagagtataGCCAATTCCCATTGACCA |
| 16 | 3639306 | CCTCTCTATGGGCAGTCGGTGATGGGGTGGTGTCCAGGAGT | CCATCTCATCCCTGCGTGTCTCCGACTCAGgcaactagttCCAAGCTTCCTGAACCAGAC |
| X | 153599770 | CCTCTCTATGGGCAGTCGGTGATCATTTTGAGGCGCGAGAA | CCATCTCATCCCTGCGTGTCTCCGACTCAGatctcgaatcCTAGTGGGGGCATTCCAA |
| X | 153599770 | CCTCTCTATGGGCAGTCGGTGATGAGGCAGGGAGCAGAGGT | CCATCTCATCCCTGCGTGTCTCCGACTCAGccaggagcgaCTCTAGGGCGCGTTTCCT |
| X | 153599770 | CCTCTCTATGGGCAGTCGGTGATCCTTTAAATGCGGGAGGAG | CCATCTCATCCCTGCGTGTCTCCGACTCAGatctccatcgTCAGCCTTTCCTCGCTCTA |
| 18 | 21453038 | CCTCTCTATGGGCAGTCGGTGATGAGCAGGAAGGGCAGGTATAA | CCATCTCATCCCTGCGTGTCTCCGACTCAGttgacgagctTCCACATAACTCGCTTGCAG |
| 18 | 21453038 | CCTCTCTATGGGCAGTCGGTGATCTGGCACAGGCTGACTCAT | CCATCTCATCCCTGCGTGTCTCCGACTCAGtactattaccGAACTGTAGCCCAGACACTGC |
| 18 | 21453038 | CCTCTCTATGGGCAGTCGGTGATGGATGCCTCCAGCAGTGA | CCATCTCATCCCTGCGTGTCTCCGACTCAGcgtcctggacACAAAGCTGGAAACTCTTCCCTA |
| X | 153587777 | CCTCTCTATGGGCAGTCGGTGATAGCCTCATAAGGGATGTACTCG | CCATCTCATCCCTGCGTGTCTCCGACTCAGctcggcgcttCCAACAAGCCCAACAAGTTC |
| X | 153587777 | CCTCTCTATGGGCAGTCGGTGATCCTTGAAAGGACTGCCTGAG | CCATCTCATCCCTGCGTGTCTCCGACTCAGgatacgtaagGAATGACCGGCTGTCTGTTT |
| X | 153587777 | CCTCTCTATGGGCAGTCGGTGATCTCCTCACCTGGCACTTGAT | CCATCTCATCCCTGCGTGTCTCCGACTCAGctcggattaaAAAGTGGCACCACCAACAA |
| 19 | 44571260 | CCTCTCTATGGGCAGTCGGTGATAGAGCCCACACAGGAGAGAG | CCATCTCATCCCTGCGTGTCTCCGACTCAGttggattcgtCTTGTAGCGCTTCCCACAGT |
| 19 | 44571260 | CCTCTCTATGGGCAGTCGGTGATATCAGCGAGTCCACACTGG | CCATCTCATCCCTGCGTGTCTCCGACTCAGccgtccgctaAGCTTCTTTCCACAATCCTCA |
| 19 | 44571260 | CCTCTCTATGGGCAGTCGGTGATTTGAATCATAAGAGACTCCATTGC | CCATCTCATCCCTGCGTGTCTCCGACTCAGgcgattgcaaCTGTACCCCATAAATATGTACAACACT |
| X | 153579431 | CCTCTCTATGGGCAGTCGGTGATCAGGGCCTCACCTTGGTC | CCATCTCATCCCTGCGTGTCTCCGACTCAGccatgcataaCGCCAGATGGGTAAGTGC |
| X | 153579431 | CCTCTCTATGGGCAGTCGGTGATCTGTGACATAGCACTCCTCCAG | CCATCTCATCCCTGCGTGTCTCCGACTCAGtaattgcaatTGCAAATCAGTGGCTCTCC |
| X | 153579431 | CCTCTCTATGGGCAGTCGGTGATAGGCTGGCTGGTTGACCT | CCATCTCATCCCTGCGTGTCTCCGACTCAGacgactccaaCTCCCTTCCTGCCACCTG |
| 12 | 46321441 | CCTCTCTATGGGCAGTCGGTGATAATCACACTCCATAGGTATCATTTCA | CCATCTCATCCCTGCGTGTCTCCGACTCAGatcatgcagaACATGTGATACTTTTGGGAATGAAG |
| 12 | 46321441 | CCTCTCTATGGGCAGTCGGTGATTTCATTCATTTGTTTAAGATCAGCA | CCATCTCATCCCTGCGTGTCTCCGACTCAGaactcctaatCTTCTGAACACCAAATTGGAAA |
| 12 | 46321441 | CCTCTCTATGGGCAGTCGGTGATTCAATGTGTGTTTTAGGCAACTC | CCATCTCATCCCTGCGTGTCTCCGACTCAGggatattcgtTGTTAAGAGCCCAGAGGTTCA |
| X | 153594210 | CCTCTCTATGGGCAGTCGGTGATAGGGGGACATGCAAGACA | CCATCTCATCCCTGCGTGTCTCCGACTCAGtcggatgactGGGGCCCCTACTCTTTGA |
| X | 153594210 | CCTCTCTATGGGCAGTCGGTGATGCGAGCTCTTCCGAAGGT | CCATCTCATCCCTGCGTGTCTCCGACTCAGgacgcgcgagCTCGCAGCCCCTACACTG |
| X | 153594210 | CCTCTCTATGGGCAGTCGGTGATGTTGACCCTGTGGGCAGA | CCATCTCATCCCTGCGTGTCTCCGACTCAGgcctagacctTGACTGCCCTCTGCTGTG |
| 16 | 3639306 | CCTCTCTATGGGCAGTCGGTGATTCCTCTGGGTAGTGCAGCTT | CCATCTCATCCCTGCGTGTCTCCGACTCAGgaccaggcgaAGTGACGATGAGCAGGAGGT |
| 16 | 3639306 | CCTCTCTATGGGCAGTCGGTGATCAGAGCCGAATTCAGAAAGC | CCATCTCATCCCTGCGTGTCTCCGACTCAGgctctggcgtGCCAATTCCCATTGACCA |
| 16 | 3639306 | CCTCTCTATGGGCAGTCGGTGATGGGGTGGTGTCCAGGAGT | CCATCTCATCCCTGCGTGTCTCCGACTCAGtggtccggaaCCAAGCTTCCTGAACCAGAC |
| X | 153599770 | CCTCTCTATGGGCAGTCGGTGATCATTTTGAGGCGCGAGAA | CCATCTCATCCCTGCGTGTCTCCGACTCAGctctgcgtctCTAGTGGGGGCATTCCAA |
| X | 153599770 | CCTCTCTATGGGCAGTCGGTGATGAGGCAGGGAGCAGAGGT | CCATCTCATCCCTGCGTGTCTCCGACTCAGccagaagcagCTCTAGGGCGCGTTTCCT |
| X | 153599770 | CCTCTCTATGGGCAGTCGGTGATCCTTTAAATGCGGGAGGAG | CCATCTCATCCCTGCGTGTCTCCGACTCAGggaaggttgcTCAGCCTTTCCTCGCTCTA |
| 18 | 21453038 | CCTCTCTATGGGCAGTCGGTGATGAGCAGGAAGGGCAGGTATAA | CCATCTCATCCCTGCGTGTCTCCGACTCAGtaacggtacgTCCACATAACTCGCTTGCAG |
| 18 | 21453038 | CCTCTCTATGGGCAGTCGGTGATCTGGCACAGGCTGACTCAT | CCATCTCATCCCTGCGTGTCTCCGACTCAGctcgctcatgGAACTGTAGCCCAGACACTGC |
| 18 | 21453038 | CCTCTCTATGGGCAGTCGGTGATGGATGCCTCCAGCAGTGA | CCATCTCATCCCTGCGTGTCTCCGACTCAGactccaaggcACAAAGCTGGAAACTCTTCCCTA |
| X | 153587777 | CCTCTCTATGGGCAGTCGGTGATAGCCTCATAAGGGATGTACTCG | CCATCTCATCCCTGCGTGTCTCCGACTCAGgagctgctatCCAACAAGCCCAACAAGTTC |
| X | 153587777 | CCTCTCTATGGGCAGTCGGTGATCCTTGAAAGGACTGCCTGAG | CCATCTCATCCCTGCGTGTCTCCGACTCAGcgttgaggccGAATGACCGGCTGTCTGTTT |
| X | 153587777 | CCTCTCTATGGGCAGTCGGTGATCTCCTCACCTGGCACTTGAT | CCATCTCATCCCTGCGTGTCTCCGACTCAGttctggatccAAAGTGGCACCACCAACAA |
| 19 | 44571260 | CCTCTCTATGGGCAGTCGGTGATAGAGCCCACACAGGAGAGAG | CCATCTCATCCCTGCGTGTCTCCGACTCAGccggattccaCTTGTAGCGCTTCCCACAGT |
| 19 | 44571260 | CCTCTCTATGGGCAGTCGGTGATATCAGCGAGTCCACACTGG | CCATCTCATCCCTGCGTGTCTCCGACTCAGtccatcgcttAGCTTCTTTCCACAATCCTCA |
| 19 | 44571260 | CCTCTCTATGGGCAGTCGGTGATTTGAATCATAAGAGACTCCATTGC | CCATCTCATCCCTGCGTGTCTCCGACTCAGttacttctcaCTGTACCCCATAAATATGTACAACACT |
