# Supplementary figures and images for: MIPP-Seq: ultra-sensitive rapid detection and validation of low-frequency mosaic mutations
Source: BMC Med Genomics. 2021 Feb 12;14:47. doi: 10.1186/s12920-021-00893-3 (PMC7881461; doi:10.1186/s12920-021-00893-3)

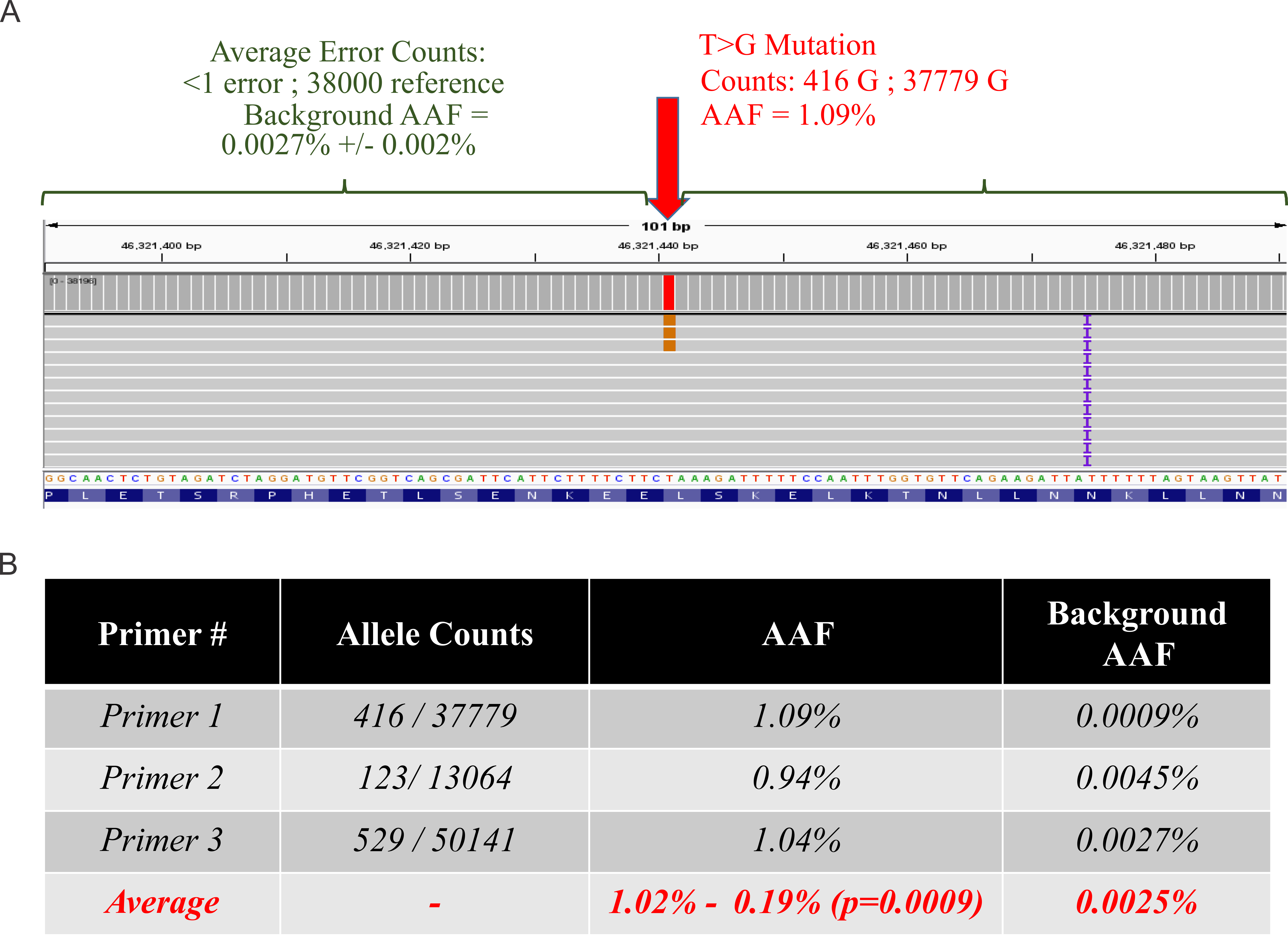

Supplement: Supplementary file 2 — Additional file 2: Fig S1. Variant allelic fraction assessment across multiple primers. A) The AAF of the targeted mutation is compared to the background error rate of 50nts flanking each side of the mutation and B) the assessed rates are averaged across all unique primers for the mutation. [file 12920_2021_893_MOESM2_ESM.tif]

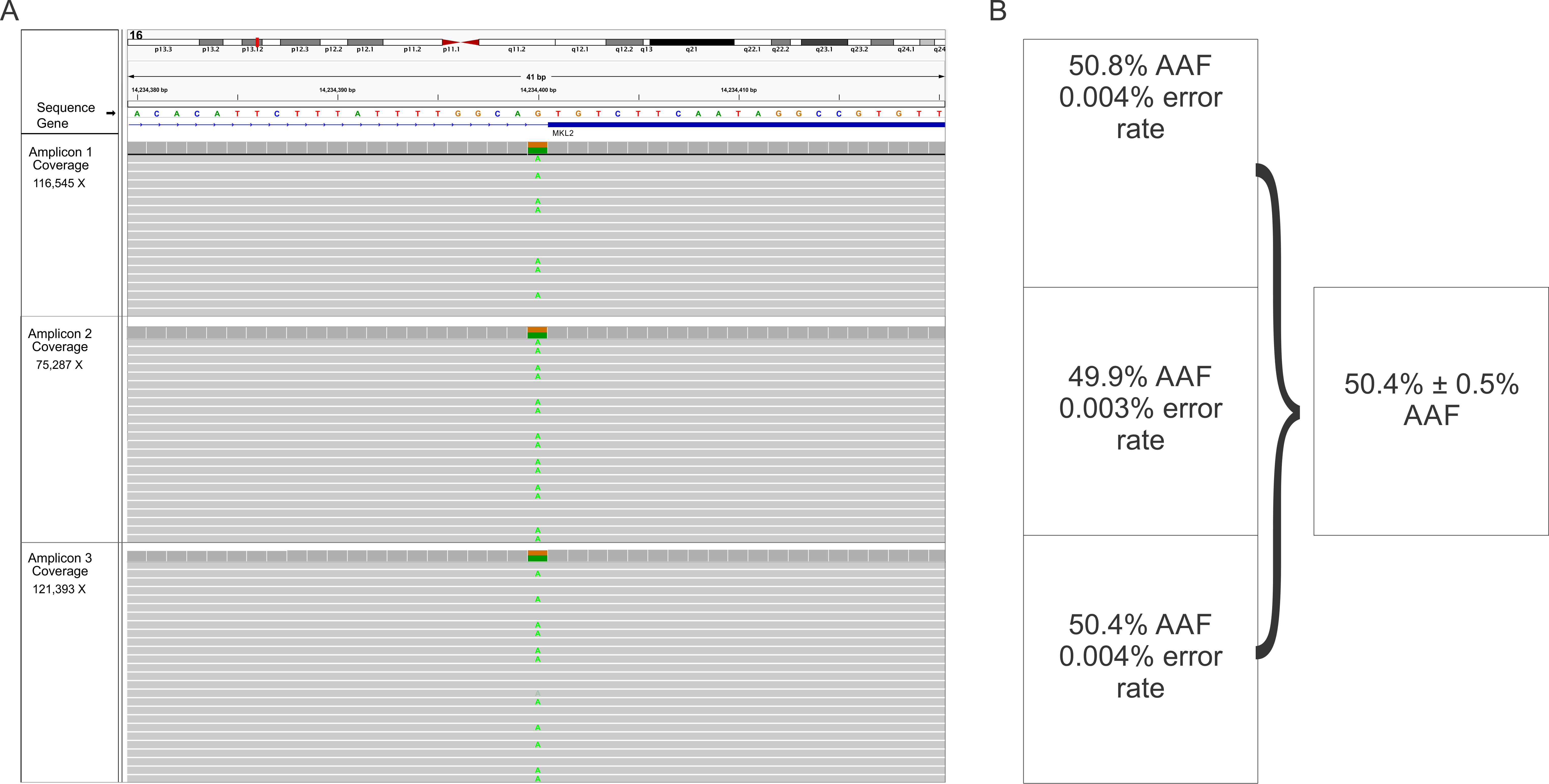

Supplement: Supplementary file 3 — Additional file 3: Fig S2. Example of validated heterozygous germline mutation. The targeted SNV was identified in A) sequencing reads for all 3 unique primers, allowing for B) the measured AAF of 50%. [file 12920_2021_893_MOESM3_ESM.tif]

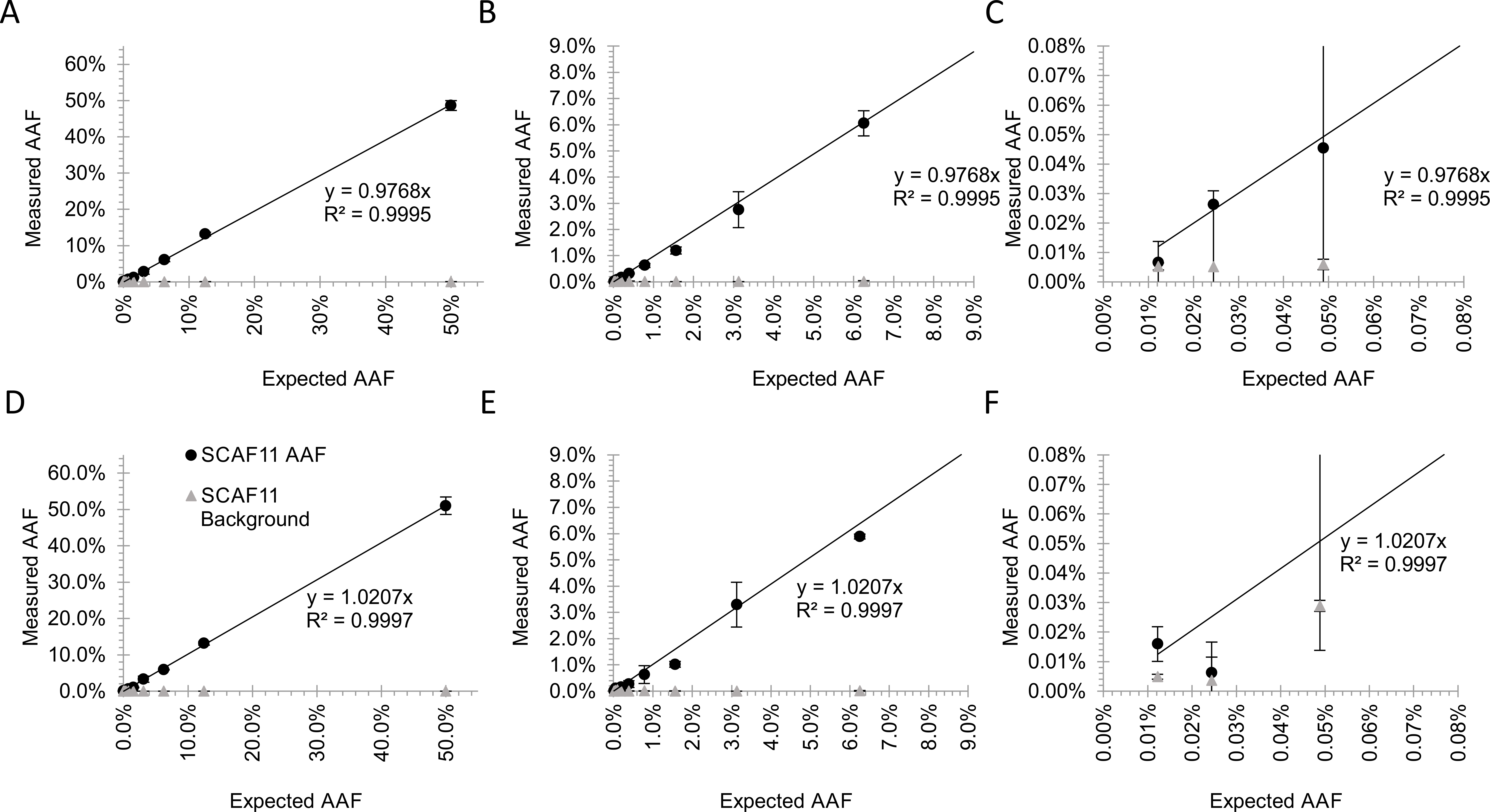

Supplement: Supplementary file 4 — Additional file 4: Fig S3. Impact on sensitivity for reduced PCR DNA input for Mutation 2. Sensitivity to measure the AAF and background error through a dilution curve of a polymorphism (Mutation 2) using A) 50ng 0.01% to 50% AAF and data subsets with AAFs B) less than 9% and C) less than 0.08%. Reduction of DNA input to 25ng with D) 0.01% to 50% AAF and data subsets with AAFs E) less than 9% and F) less than 0.08%. [file 12920_2021_893_MOESM4_ESM.tif]

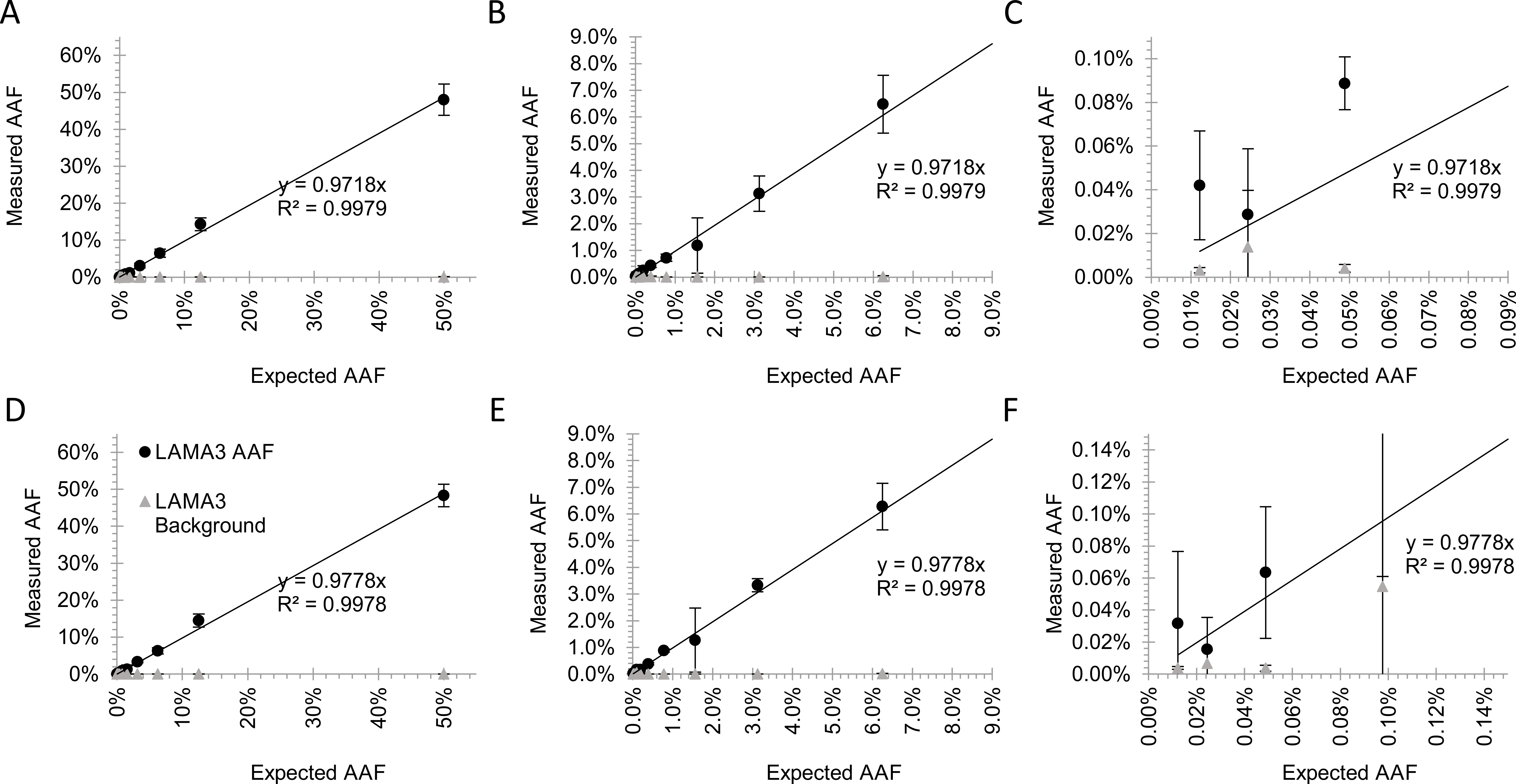

Supplement: Supplementary file 5 — Additional file 5: Fig S4. Impact on sensitivity for reduced PCR DNA input for Mutation 3. Sensitivity to measure the AAF and background error through a dilution curve of a polymorphism (Mutation 3) using A) 50ng 0.01% to 50% AAF and data subsets with AAFs B) less than 9% and C) less than 0.08%. Reduction of DNA input to 25ng with D) 0.01% to 50% AAF and data subsets with AAFs E) less than 9% and F) less than 0.08%. [file 12920_2021_893_MOESM5_ESM.tif]

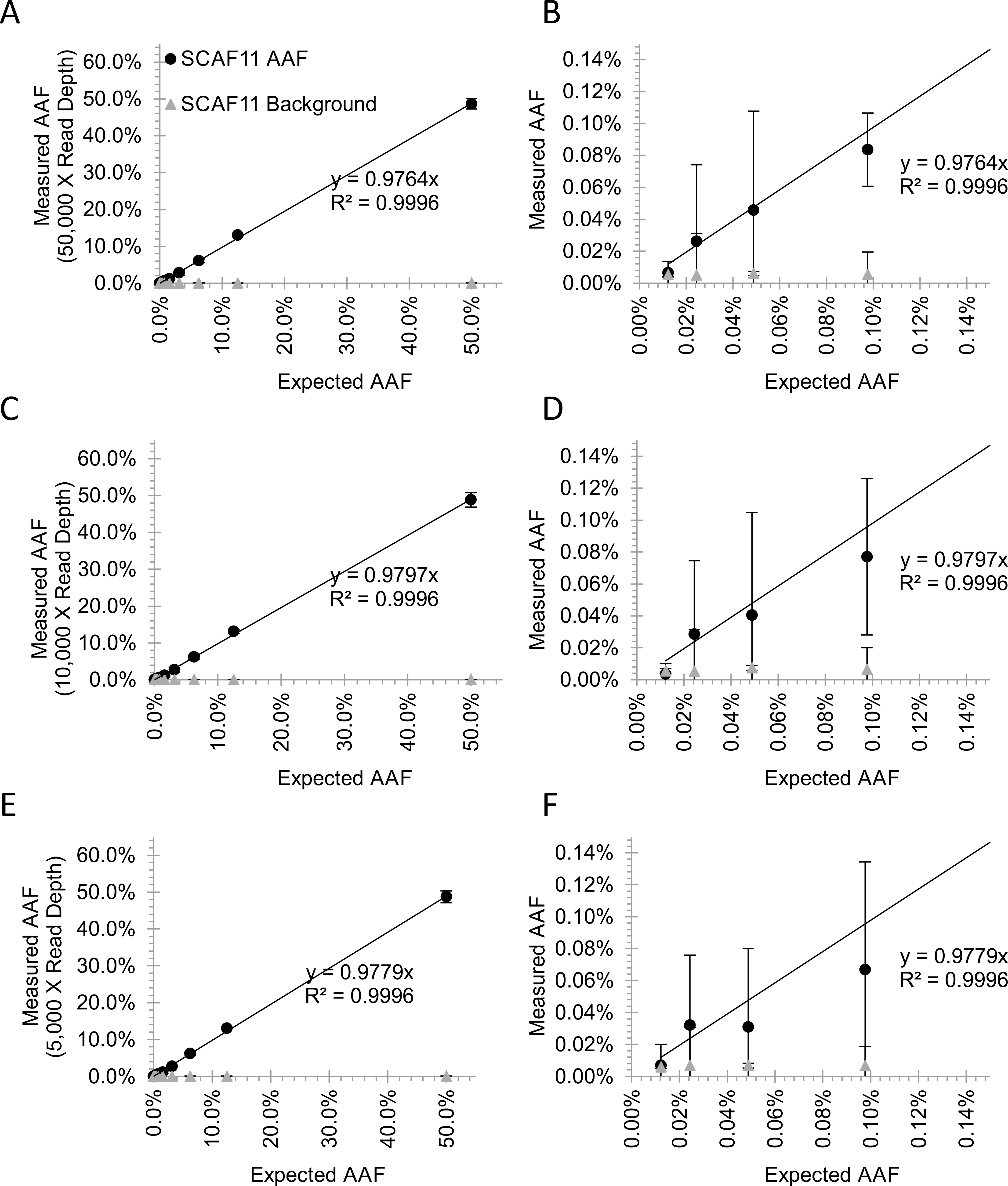

Supplement: Supplementary file 6 — Additional file 6: Fig S5. Impact of read depth on sensitivity of AAF assessments for Mutation 2. Reduction of initial maximum read depth from 50,000X for detection of alleles from A) 50% to B) 0.025% to C) & D) 10,000X and E) & F) 5,000X. [file 12920_2021_893_MOESM6_ESM.tif]

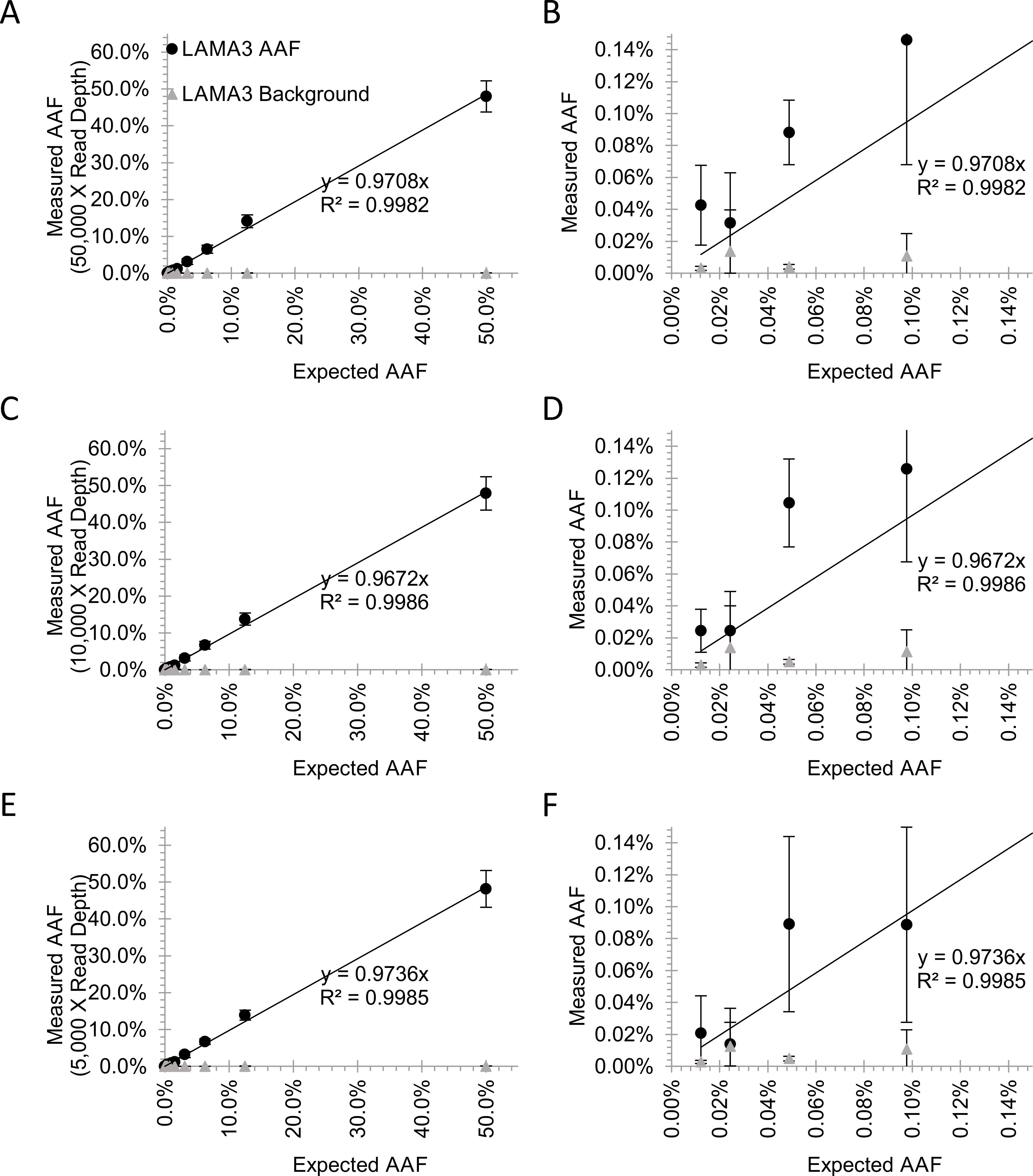

Supplement: Supplementary file 7 — Additional file 7: Fig S6. Impact of read depth on sensitivity of AAF assessments for Mutation 3. Reduction of initial maximum read depth from 50,000X for detection of alleles from A) 50% to B) 0.025% to C) & D) 10,000X and E) & F) 5,000X. [file 12920_2021_893_MOESM7_ESM.tif]

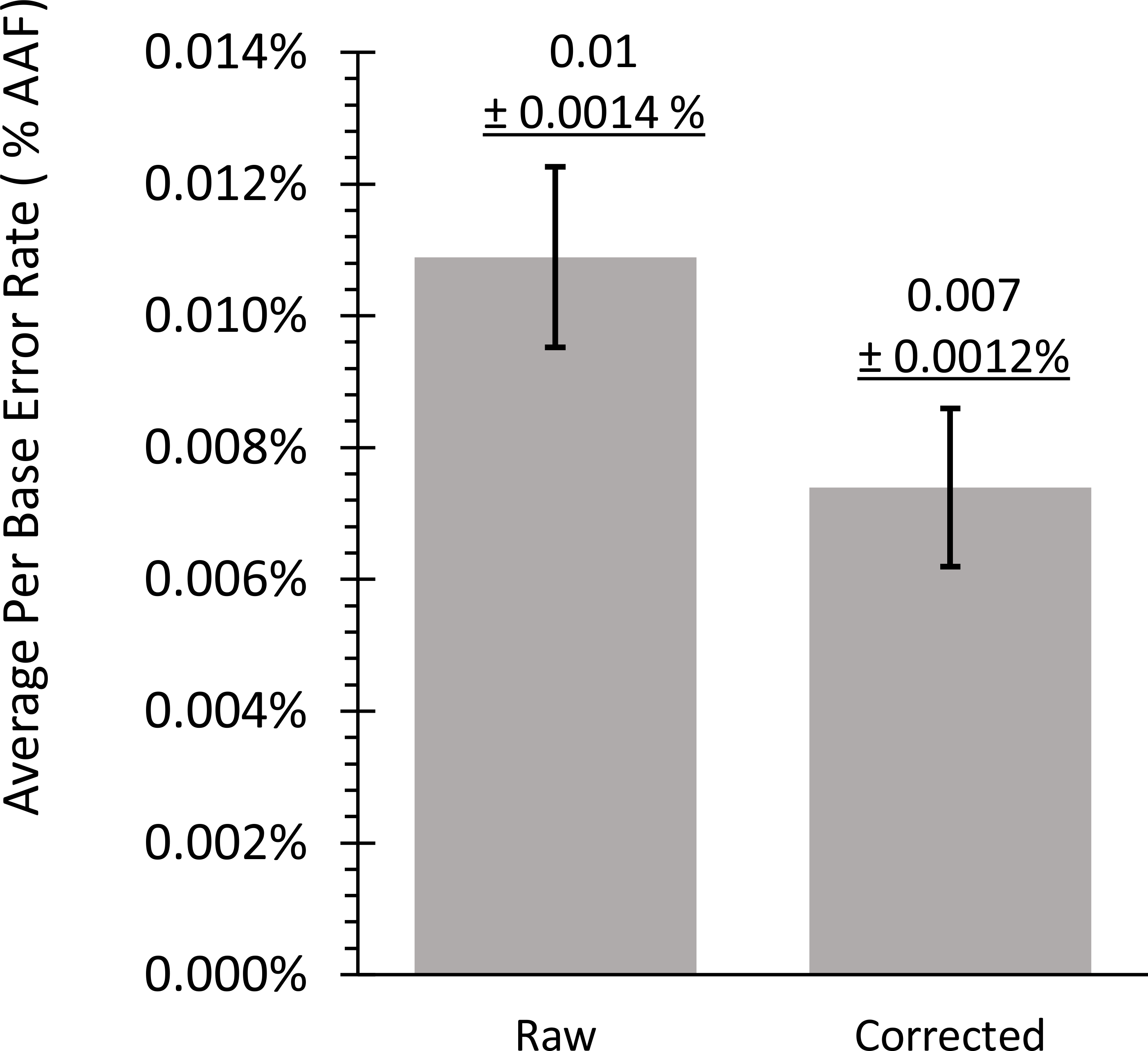

Supplement: Supplementary file 8 — Additional file 8: Fig S7. Reduction of background average per base error rate through error correction. [file 12920_2021_893_MOESM8_ESM.tif]

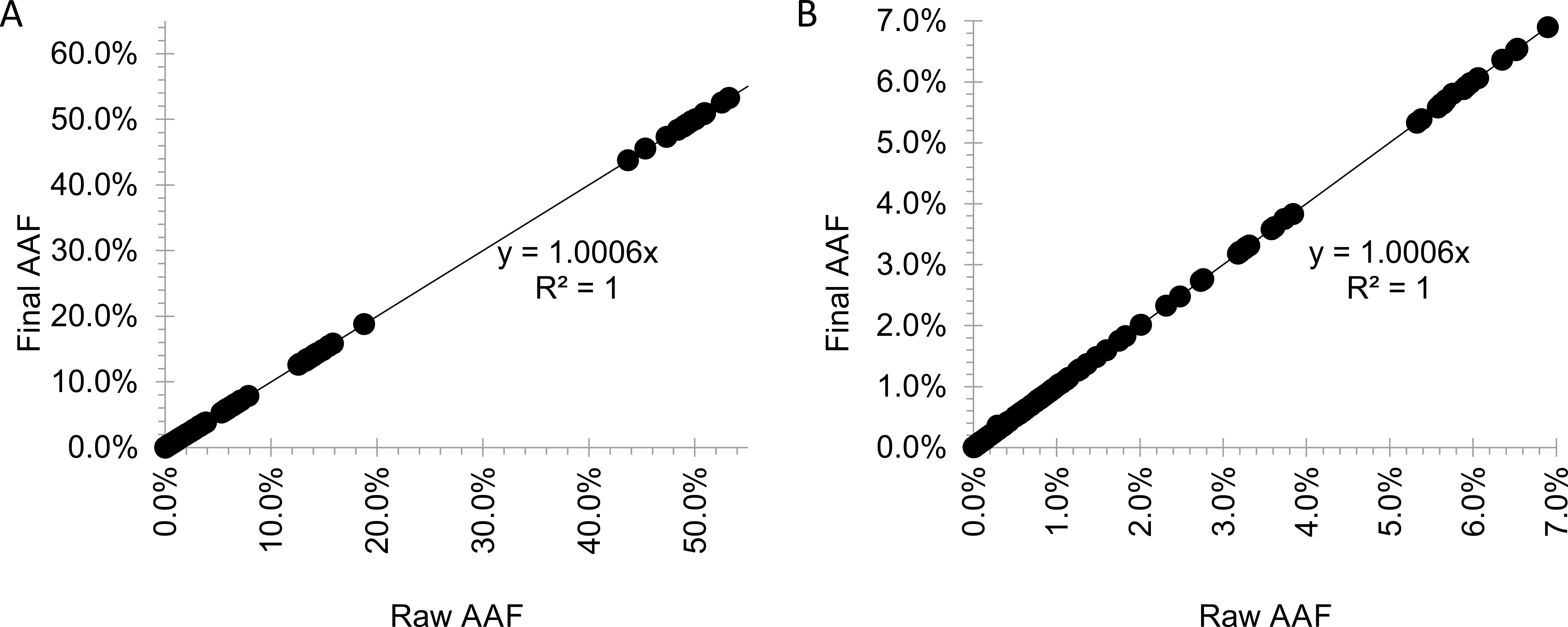

Supplement: Supplementary file 9 — Additional file 9: Fig S8. Strong correlation of AAFs before and after error correction by Pollux algorithm. A) Correlation of raw AAFs, those detected from data prior to error correction, with the final AAFs (i.e., post-error correction), with B) a subset of data below 7% AAF also showing a strong correlation. [file 12920_2021_893_MOESM9_ESM.tif]

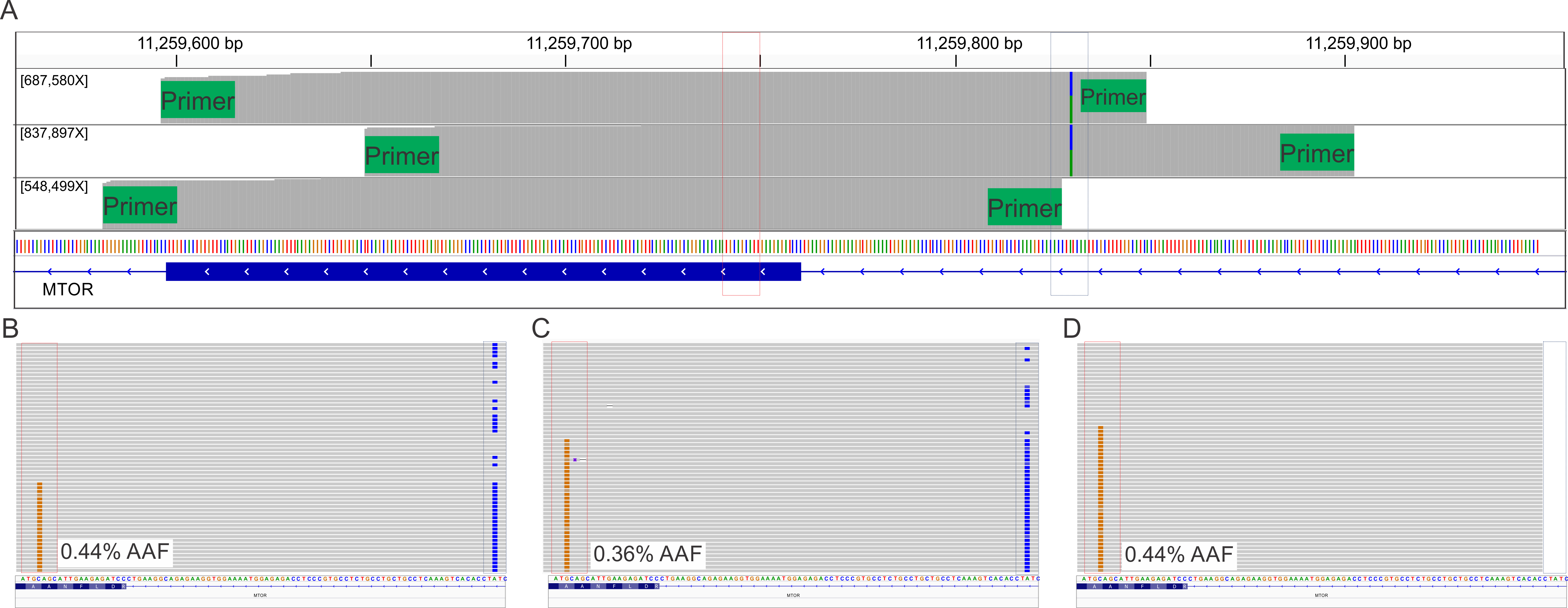

Supplement: Supplementary file 10 — Additional file 10: Fig S9. Mosaic mutation in cis with germline polymorphism. A) Analysis of a mosaic point mutation revealed a germline polymorphism in 2 of the 3 amplicons. Amplicons 1 and 2 (B &C) covered both the germline and mosaic event and confirmed the cis arrangement, while the 3rd amplicon D) did not include the germline event. [file 12920_2021_893_MOESM10_ESM.tif]

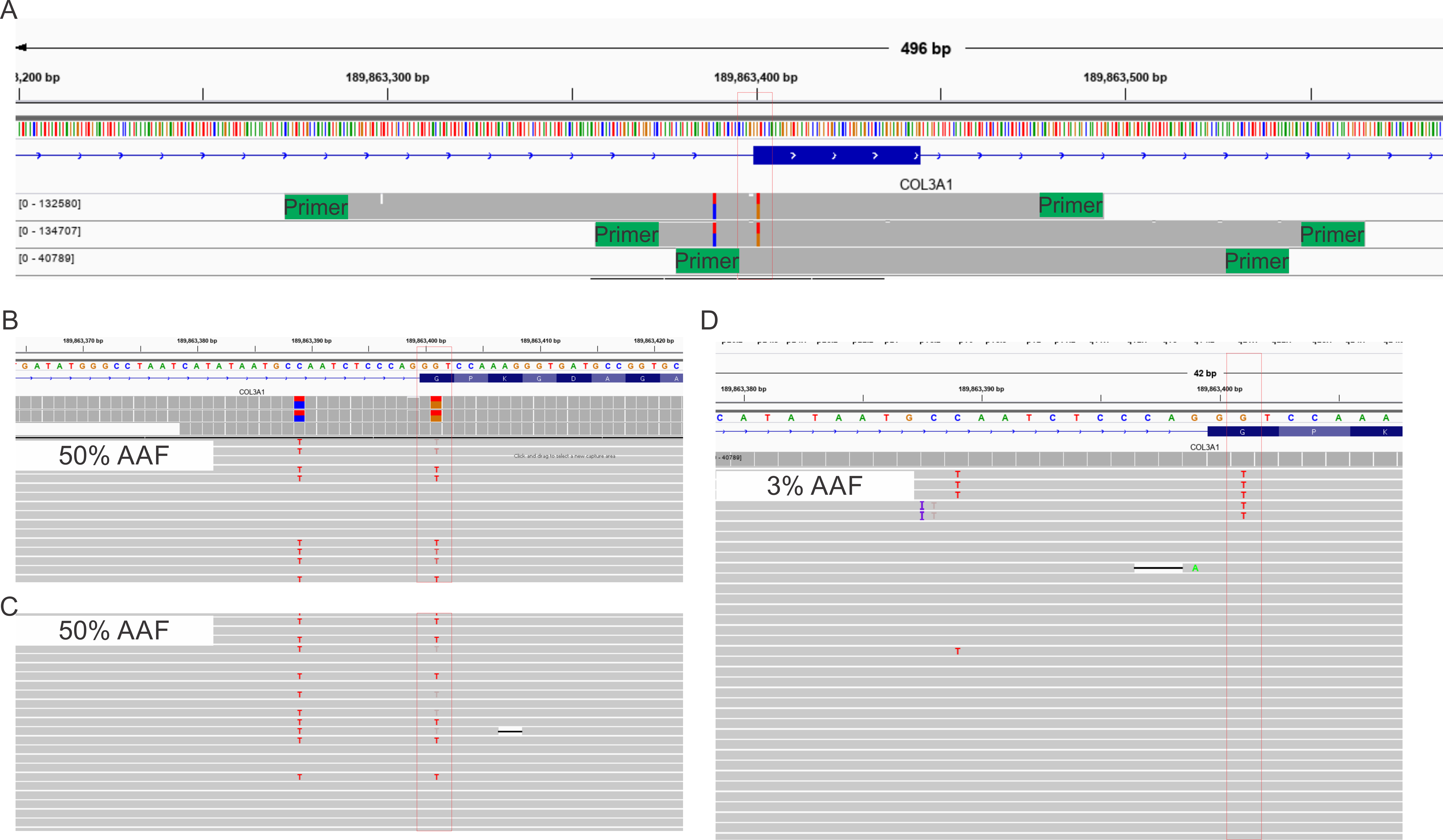

Supplement: Supplementary file 11 — Additional file 11: Fig S10. Detection of allele dropout masking germline event. A germline mutation was targeted by A) 3 unique sets of primers. Mapped sequencing data for B) amplicon 1 and C) amplicon 2 yielded the expected 50% AAF mutation and identified a common polymorphism nearby and located in the binding site of the D) third primer, interfering with binding and resulting in a dramatically skewed AAF. [file 12920_2021_893_MOESM11_ESM.tif]

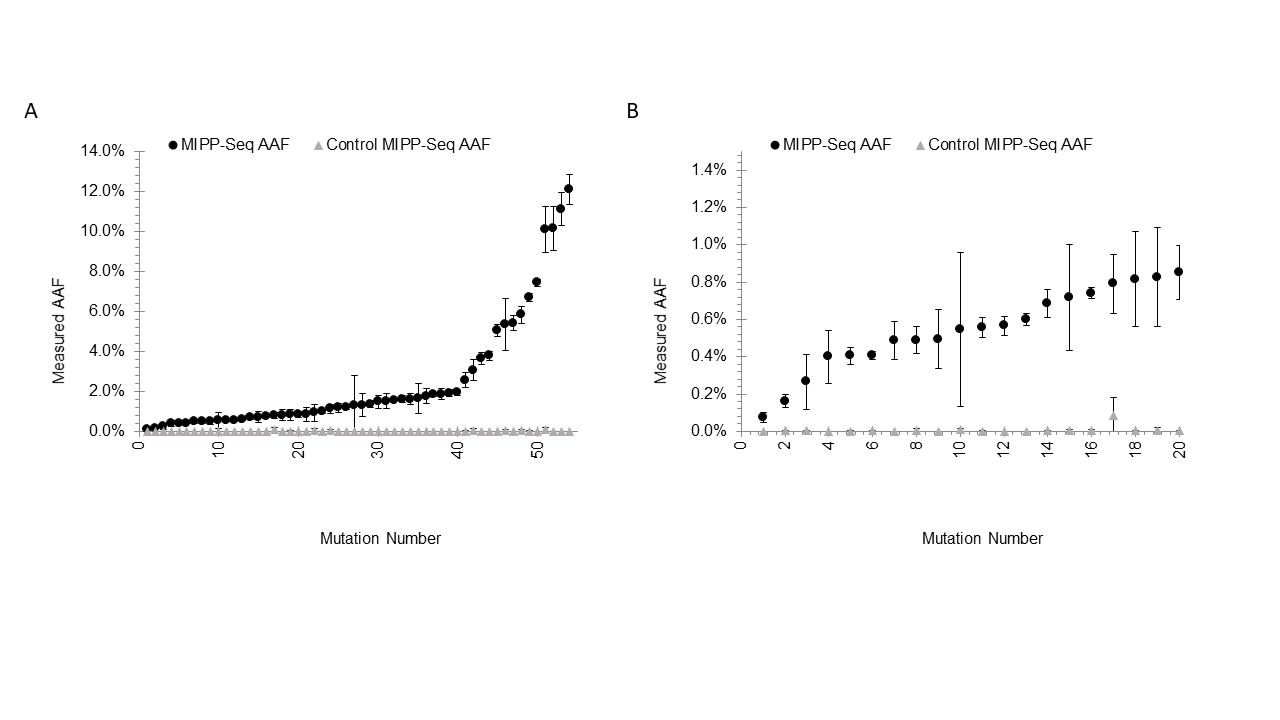

Supplement: Supplementary file 12 — Additional file 12: Fig S11. Comparison of AAFs detected for indels in cases vs controls. Indels were validated using MIPP-seq on the case DNA sample contained a suspected indel and a different control DNA sample lacking the indel. All indels validated by MIPP-seq exhibited a significantly higher AAF in the case (black filled circles) vs control DNA (grey triangles) A) and B). [file 12920_2021_893_MOESM12_ESM.tif]
